# LPA receptor activity is basal specific and coincident with early pregnancy and involution during mammary gland postnatal development

**DOI:** 10.1038/srep35810

**Published:** 2016-11-03

**Authors:** Deanna Acosta, Susmita Bagchi, Pilib Ó Broin, Daniel Hollern, Silvia E. Racedo, Bernice Morrow, Rani S. Sellers, John M. Greally, Aaron Golden, Eran Andrechek, Teresa Wood, Cristina Montagna

**Affiliations:** 1 Department of Genetics, Albert Einstein College of Medicine, Bronx, NY, 10461, USA; 2 Department of Pathology, Albert Einstein College of Medicine, Bronx, NY, 10461, USA; 3 Department of Physiology, Michigan State University, East Lansing, MI, 48824, USA; 4 Department of Neurology and Neuroscience, Rutgers New Jersey Medical School, Newark, NJ, 07101, USA

## Abstract

During pregnancy, luminal and basal epithelial cells of the adult mammary gland proliferate and differentiate resulting in remodeling of the adult gland. While pathways that control this process have been characterized in the gland as a whole, the contribution of specific cell subtypes, in particular the basal compartment, remains largely unknown. Basal cells provide structural and contractile support, however they also orchestrate the communication between the stroma and the luminal compartment at all developmental stages. Using RNA-seq, we show that basal cells are extraordinarily transcriptionally dynamic throughout pregnancy when compared to luminal cells. We identified gene expression changes that define specific basal functions acquired during development that led to the identification of novel markers. Enrichment analysis of gene sets from 24 mouse models for breast cancer pinpoint to a potential new function for insulin-like growth factor 1 (Igf1r) in the basal epithelium during lactogenesis. We establish that β-catenin signaling is activated in basal cells during early pregnancy, and demonstrate that this activity is mediated by lysophosphatidic acid receptor 3 (Lpar3). These findings identify novel pathways active during functional maturation of the adult mammary gland.

The adult mammary gland is a complex tissue composed of many different cell types that function together to provide nutrients in the form of milk proteins and lipids, as well as protective immune factors for the offspring. The mammary gland contains two major tissue compartments, the epithelium and the stroma within the mammary fat pad. Luminal cells are the major component of the epithelial layer. They surround the duct, undergoing differentiation into milk-producing alveoli during pregnancy. The basal layer of the epithelium, composed primarily of myoepithelial cells, is a meshwork of cells that enclose the luminal cells and contract during lactation to assist in the secretion of milk. These cells also contribute to the synthesis of the basement membrane, which surrounds the epithelial compartment^1^. Communication between and within the cellular compartments is essential for the functional development and differentiation of the mammary gland^2^,^3^,^4^,^5^,^6^,^7^,^8^.

The functional development of the mammary gland primarily occurs postnatally. At birth only a rudimentary gland is present^9^. Proliferation of the epithelial cells and invasion into the mammary fat pad occurs at puberty with the ducts reaching the end of the fat pad, shaping the mature gland^10^,^11^. Once pregnancy begins, the luminal epithelial cells proliferate, producing tertiary branches, whereby they differentiate into milk-producing alveolar cells^12^,^13^. The first stage of lactogenesis occurs during late pregnancy when lipid droplets form and milk proteins are produced and secreted. The second stage is characterized by the abundant milk secretion that occurs after parturition, when mature alveolar cells produce and secrete milk into the lumen of the alveoli^12^,^14^. It is only at this stage that the gland reaches a fully differentiated state^15^. After lactation, involution of the mammary epithelium begins resulting in the tightly regulated death of alveolar cells and extensive tissue remodeling to revert the gland to a pre-pregnancy-like state.

The current knowledge of the functional differentiation and development of the mammary gland is largely based on studies of the luminal epithelial population because luminal cells (i) are the most prevalent cell type in the mammary gland, especially during pregnancy and lactation; (ii) produce milk proteins and lipids, and therefore are accountable for the major function of the mammary gland; (iii) are the origin of the most common and malignant breast cancer subtypes^16^,^17^,^18^,^19^,^20^. Recent interest in basal epithelial cells has heightened due mainly to the discoveries that this population regulates the structural integrity of the epithelial compartment, communicates with luminal cells to regulate ductal outgrowth and branching morphogenesis during puberty and comprises a minor population of mammary stem cells^6^,^8^,^21^,^22^,^23^,^24^,^25^,^26^,^27^,^28^,^29^. Recent evidence reveals that the basal compartment provides signals to coordinate the functional differentiation of luminal progenitor cells during lactogenesis^30^.

The genes and signaling pathways driving development of the mammary gland have been extensively characterized^31^,^32^,^33^,^34^,^35^,^36^,^37^. These studies have been fundamental to identify pathways governing the various phases of mammary gland development. However, a major limitation of these studies is the use of combined RNA from all cell subtypes present in the adult mammary gland. The results most likely reflect the transcriptional profile of the dominant cell type, the luminal epithelial cells, during mammary gland development. The basal cells are less prevalent; thus, minor development-specific gene expression changes in this subtype may remain undetected. Together with luminal cells, the basal epithelial population undergoes significant changes at the gene expression level when exposed to the ovarian hormones 17β-estradiol and progesterone^7^,^38^. Gene expression analysis of four different human and mouse mammary epithelial cell populations (mammary stem/basal cells, committed luminal progenitor, mature luminal and stromal cell) revealed that the basal population contains the largest number of conserved genes between the two species providing insight into conserved lineage-specific pathways functional in the adult mammary gland^39^. These studies also validated the use of mouse models for studying gene expression profiles in enriched populations during normal mammary gland development. However, a detailed characterization at the gene expression level of enriched epithelial cell populations during all stages of postnatal mammary gland development remains to be done.

The work presented here transcriptionally profiles enriched basal populations during eight different stages of murine postnatal mammary gland development compared to enriched luminal epithelial cells. Our results uncover an unexpected dynamic transcriptional activity in basal cells that led us to identify an expression profile that defines the specific functions acquired by these cells during mammary gland differentiation. We discovered novel markers associated with known basal functional pathways. Additionally, gene set enrichment analysis of the spatio-temporal gene set with a gene set of 24 mouse models for breast cancer led us to identify a potential new function for insulin-like growth factor 1 (Igf1r) in the basal epithelium during lactogenesis. Finally, we established that exposure of basal cells to lysophosphatidic acid (LPA) triggers a morphological switch in these cells that is associated with β-catenin signaling activation and mediated by lysophosphatidic acid receptor 3 (Lpar3). We propose that during early pregnancy, canonical Wnt signaling is initiated in the basal epithelial cells and that this activity is mediated by Lpar3 signaling. These findings greatly improve the current knowledge of basal cell function during mammary gland development, and provide novel markers for functional validation in normal mammary gland development and breast tumorigenesis.

## Results

### Generation of CD24^+^ CD29^lo^ and CD24^+^ CD29^hi^ RNAseq datasets across murine mammary postnatal development

To characterize the transcriptional activity of basal cells relative to the luminal population during pregnancy, lactation and involution we first isolated luminal and basal epithelial cells during postnatal development^27^,^28^,^29^,^39^,^40^,^41^,^42^,^43^,^44^,^45^,^46^. Across all time points, the mammary epithelial cells consistently separated into two distinct epithelial cell populations, Lin^−^CD24^+^ CD29^lo^ and Lin^−^CD24^+^ CD29^hi^ (referred to as the luminal and basal subtypes, respectively) (Supplementary Figure S1). We worked under the assumption that the expression of the markers used for sorting was not altered during development. As an initial technical validation of luminal and basal cell enrichment, we re-sorted 1,000 cells from each population and confirmed low cross contamination (<2%) between the Lin^−^CD24^+^ CD29^lo^ and Lin^−^CD24^+^ CD29^hi^ enriched cells (Supplementary Figure S1). Overall, our experimental approach allowed for the collection of a sample set of enriched luminal and basal cells from the murine mammary epithelium during eight time points of postnatal development.

**Figure 1:**
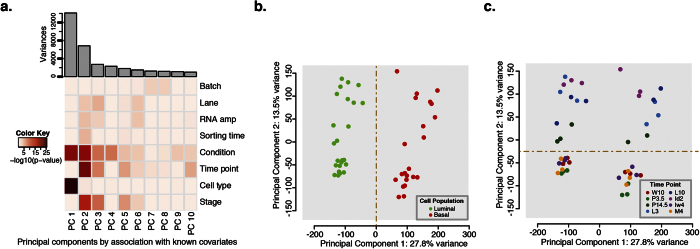
Analysis of the transcriptional variation between samples. Principal component analysis (PCA) was conducted to assess the variability between the samples based on their transcriptional profile. (**a**) Scree plot of the amount of variance associated with each principal component (top). Heatmap of the p-values of the association between biological (Condition, Time point, Cell type and Stage) and technical (Batch, Lane, RNA amp, Sorting time) covariates (rows) and the 10 major principal components. Dark red signifies a highly significant association (p-value = 10^−25^) and white represents less significant associations (p-value = 1). (**b,c**) Plots of the first two major principal components colored by cell population and time point, respectively. (**b**) Cell populations: green = luminal and red = basal (**c**) Time points: dark red = week 10 (W10), light green = pregnancy day 3.5 (P3.5), dark green = pregnancy day 14.5 (P14.5), light blue = lactation day 3 (L3), dark blue = lactation day 10 (L10), light purple = involution day 2 (Id2), purple = involution week 4 (Iw4) and orange = month 4 (M4). See also Supplementary Figure S2.

To identify cell subtype specific gene expression changes occurring during postnatal mammary gland development, we performed transcriptomic analysis using RNA-sequencing on enriched basal cell populations and compared the developmental profile to matched luminal cells in three biological sets. Sample outliers were identified by comparing the Pearson correlation coefficients between samples and sample-sample variability as determined by principal component analysis (PCA). Subsequent analyses excluded the identified outlier (Supplementary Figure S2). We conducted a preliminary technical validation using luminal cells from the early pregnancy and lactation time points and found that 78.5% of the genes tested by RT-PCR validate the RNA-seq findings (Supplementary Figure S2). To discern the contribution of technical versus biological variation to global transcriptomic changes, we conducted a PCA on the normalized data set. We used an approach adapted from Teschendorff *et al.*^47^, in which we calculated a p-value for the association between each covariate and a principal component (PC) using analysis of variance (ANOVA) linear modeling. Since each time point and replicate were sorted independently over a course of several months, we wanted to ensure that this variable did not introduce uncontrolled noise to the dataset and included it as a technical covariate (labeled as Sorting time on the heatmap, Fig. 1a). The first two principal components embodied most of the variability (41.3%) observed between the samples. Cell subtype differences were substantially represented in the first principal component with a p-value less than 2.9 × 10^−26^ (Fig. 1a) and contributed, as expected, to the most difference. The other variables of biological interest, cell population, specific developmental time point and pregnancy stage, were significantly associated with PC1 and PC2 with p-values less than 2.5 × 10^−15^ (Fig. 1a). All technical covariates analyzed displayed an insignificant contribution to PC1 (p-values > 0.1). The use of different sequencing lanes, RNA amplification batch and variations due to sorting times were slightly more associated with PC2 (p-values 3.5 × 10^−4^, 2.4 × 10^−3^ and 7.4 × 10^−4^, respectively), though these associations were minor relative to condition, time point and stage (p-values < 2.5 × 10^−15^) (Fig. 1a, Supplementary Figure S2). We therefore concluded that expression changes were associated with biological functions of mammary gland development, rather than technical variation. The first component differentiates the luminal and basal epithelial cell populations, while the second component was primarily driven by stage-specific differences (Fig. 1b,c).

### Gene set expression of luminal and basal specific markers remains constant in CD24^+^ CD29^lo^ and CD24^+^ CD29^hi^ enriched populations though pregnancy and lactation and defines luminal and basal cell subtypes

**Figure 2:**
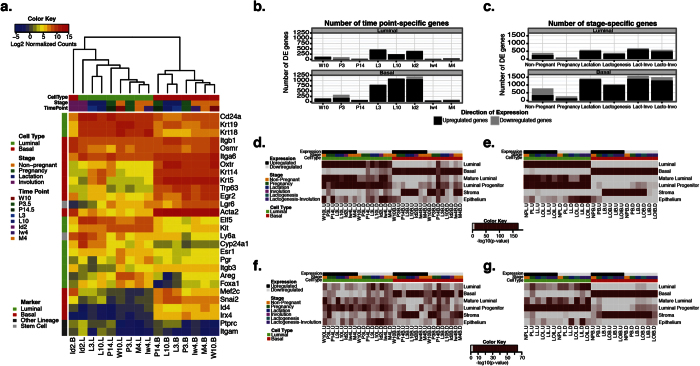
Enrichment of luminal and basal populations during postnatal mammary gland development. (**a**) Supervised heatmap clustering of luminal (green columns) and basal (red columns) epithelial cell populations based on the log2 transformed expression of known luminal (green rows), basal (red rows) and stem cell/other lineage (grey rows) markers. (**b,c**) Barplots of the total number of time point and stage specific genes, respectively, in the luminal (top) and basal (bottom) populations. Lactogenesis (P14.5, L3 and L10), lact-invo (lactation-involution, L3, L10 and Id2) and lacto-invo (lactogenesis-involution, P14.5, L3, L10 and Id2). (**d–g**) Heatmap representation of the enrichment of time point and stage specific gene sets (**b** and **c**, respectively) in gene sets from enriched mature luminal, luminal progenitor, basal and stroma populations isolated from the mouse (**d,e**) and human (**f,g**). Stages are abbreviated as non-pregnant (NP), pregnancy (P), lactation (L), lactogenesis (LO), lactation-involution (LI) and lactogenesis-involution (LOI). The stage abbreviations are categorized as either luminal (L) or basal (B) and up- (.U) or downregulated (.D). The luminal population gene set was obtained by comparing both the mature luminal and luminal progenitor populations to the basal population. The epithelial population gene set was obtained by comparing the combined mature luminal, luminal progenitor and basal populations to the stromal population. Dark red blocks indicate a statistically significant enrichment (p < 0.01). See also Supplementary Figures S2 and S3. For a comprehensive list of differentially expressed genes identified, see Supplementary Table S2.

To our knowledge, our dataset is the first of its kind and therefore there are no known, distinct luminal and basal developmental markers. Consequently we manually curated a list of markers commonly used for FACS enrichment of mammary cells^27^,^28^,^42^, luminal and basal mammary epithelial cell markers^27^,^39^,^42^,^43^,^45^,^48^,^49^ and mammary gland development genes^12^,^50^,^51^. To ensure enrichment of the cell populations at all time points, we first conducted a supervised clustering analysis to test how the samples group relative to the expression of a curated set of luminal and basal cell markers (Fig. 2a). The samples clustered into two distinct groups based on their specific cell subtypes. Surprisingly, the early involution (Id2B) basal population clustered with, yet was still distinct from, the luminal cells. Though genes for several well characterized basal markers, *Acta2* (smooth muscle actin), *Mef2c* (myocyte enhancer factor 2c), *Snai2* (snail homolog 2) and *Lifr* (leukemia inhibitory factor receptor alpha) were maintained at a significantly higher level than in luminal cells (FDR < 0.01), other well characterized markers such as *Trp63* (tumor protein 63), *Krt5/14* (cytokeratins 5 and 14) and *Id4* (inhibitor of DNA binding 4) appear to be down regulated in this population during early involution (Fig. 2a), yet further validation would be required at the protein level. The other basal markers provided additional evidence that the basal population is enriched for basal epithelial cells, and the dynamic expression patterns during the different time points may be reflective of their function during mammary gland development. In the luminal epithelial cells, the well-characterized luminal epithelial cell marker cytokeratin 18 (*Krt18*) was highly expressed throughout development relative to the basal cells. The expression of other luminal markers varied throughout development, but they were predominantly expressed in the luminal population (Fig. 2a). As expected, the majorities of the lineage negative markers (see Supplementary Table S1) were not detectable, or were expressed at very low levels in both cell populations (Fig. 2a). Additionally, the luminal and basal markers used for sorting, *Cd24a* and *Itgb1* (CD29), were consistently highly expressed throughout development, demonstrating that we collected a population of Lin^−^CD24^+^ CD29^lo^ and Lin^−^CD24^+^ CD29^hi^ cells at all stages of pregnancy, lactation, and involution. We therefore concluded that the sorted populations consist of basal and luminal cells at all stages analyzed.

We next sought to determine if the transcriptomes of these populations reflect their known developmental functions. The initial principal component analysis demonstrated that the expression profile distinguishes the lactogenic and early involution stages from the non-pregnant and early pregnancy time points (Fig. 1c). To confirm that the variability in gene expression reflects the functional changes that occur during postnatal mammary gland development, we assessed the expression of several genes known to be involved in lactation, and cell contraction during lactation and involution of the mammary gland. We found that these genes displayed both stage and cell subtype specific expression patterns (Supplementary Figure S2). This demonstrates that our novel transcriptomic data set also reflects the functional changes that occur in luminal and basal epithelial cell populations during postnatal mammary gland development. Taken together, we have established a unique RNA-seq data set that allows for an in depth investigation of cell subtype-specific expression changes during mouse postnatal mammary gland development.

### Spatio-Temporal Transcriptomic Analysis Reveal that the Basal Epithelial Cell Population is More Transcriptionally Dynamic Relative to the Luminal Population During Lactation and Involution

We next hypothesized that basal cell transcription profiles are unique at each stage of postnatal mammary gland development. To characterize the basal epithelial cells and identify potential novel cell subtype-specific mammary gland development genes, we explored the spatio-temporal transcriptome profile of the enriched basal population relative to luminal cells. From the PCA, we determined that the developmental stages separate based on the onset of lactogenesis, represented by the late pregnancy time point in this dataset (P14.5). The non-pregnant time points cluster close together with early pregnancy (P3.5) and therefore are more tightly associated at the gene expression level than the later time points (Fig. 1c and Supplementary Figure S2). The lactogenesis and involution time points are not as strongly associated to one another (Fig. 1c), suggesting that the gene expression profile of both cell populations during lactogenesis and early involution is highly dynamic.

With this study, we aimed to assess the global transcriptome dynamics of luminal and basal epithelial cells with an emphasis on identifying novel basal specific pathways. We used ANOVA to filter for genes that were differentially expressed (DE) in at least one cell population during a minimum of one time point (FDR < 0.01), and found that 12,663 (71.9%) of the 17,606 normalized genes met this criterion (Supplementary Figure S3). To identify genes that were spatially or temporally regulated, we set a conservative, arbitrary threshold of >2-fold change and FDR < 0.01. Given that within our samples cell population and stage specific differences contributed the most to variability, we did not include the technical covariates in the linear model. We found that, of the spatially and temporally expressed genes, 8,718 (68.8%) genes were spatially DE between luminal and basal cells in at least one time point, and 9,821 (77.6%) genes were temporally DE between any two time points in either cell subtype (Supplementary Figure S3). Additionally, 50.7% of the 12,663 expressed genes were both spatially and temporally DE within the cell populations, meaning that of the spatially regulated genes, 73.6% were also temporally differentially expressed. Based on these findings, we concluded that most (95.7%) of the genes expressed in adult luminal and basal mammary epithelial cells are spatially and temporally regulated during pregnancy, lactation and involution.

To characterize the individual cell populations during the developmental time points, we focused our analysis on changes of temporal patterns within the group of spatially DE genes (Supplementary Figure S3). We defined luminal and basal specific genes as those that were upregulated in either the luminal or basal population in at least one time point during development relative to the other population (fold change ≥2, FDR < 0.01). Of the 8,718 spatially DE genes, 35.8% were luminal specific, 46.7% were basal specific and a subset of the genes (17.5%) switched between luminal and basal specificity throughout development (Supplementary Figure S3). Of the luminal specific genes, 570 (18.3%) were specific to the luminal population throughout all stages of development, and of the basal specific genes, 1,292 (31.7%) were specific to this population. These findings indicate that the majority of the differentially expressed genes in each population were not population specific across all the developmental time points analyzed and support the premise for a temporally regulated pattern.

The basis of the present characterization of the luminal and basal populations during postnatal mammary gland development was to identify genes that are time point and/or stage specific. As the primary functional and morphological changes occur in the mammary epithelium during pregnancy, lactation and involution, we expected a dynamic expression profile during these time points in both cell subtypes. We performed pairwise comparisons between each time point/stage and the remaining time points (or stages) and selected genes that were statistically significant across all comparisons (fold change ≥2 and FDR < 0.01). All genes that met this threshold are listed in Supplementary Table S2. Stages are defined in Supplemental Procedures Table 1 with the exception of lactogenesis (P14.5, L3 and L10), lactation-involution (lact-invo, L3, L10 and Id2) and lactogenesis-involution (lacto-invo, P14.5, L3, L10 and Id2). A larger number of differentially expressed genes were detected in luminal cells during late pregnancy, lactation and early involution time points (Fig. 2b, top). The stage specific temporal analysis allowed for the detection of additional novel genes that were differentially regulated across multiple time points. We found that the lactation (L3 and L10 time points) and lactatogenesis-involution (lacto-invo, P14.5, L3, L10 and Id2 time points) stages had the largest number of DE genes (Fig. 2c, top). The basal population had an overwhelmingly larger number of significant DE genes in both the time point and stage specific analysis relative to the luminal population, 3,144 versus 1,559 unique DE genes, respectively (Fig. 2b,c). Most of the changes in the basal population occurred in the P3.5, L3, L10 and Id2 time points, and during the lactation and lactogenesis-involution stages (Fig. 2b,c, bottom).

To biologically validate our dataset, we assessed the enrichment of the spatio-temporal gene set (Fig. 2b,c) in a published dataset of murine and human luminal and basal specific genes. We used a published expression data set of sorted mature luminal, luminal progenitor, mammary stem cell enriched (our basal population) and stroma cells from adult mouse and human mammary tissue (GEO series GSE19446 and GSE16997)^39^. We found a significant (p < 0.01) enrichment of the Lim *et al.* mouse luminal and basal genes in our luminal and basal gene sets, respectively (Fig. 2d,e). When we tested for conservation in human, we found a significant enrichment of the luminal populations in some of our luminal gene sets, and a more consistent, significant enrichment in the basal population as previously observed^39^ (Fig. 2f,g). We also observed a significant enrichment of stromal genes in our basal gene sets. This was consistent with the published data set, where of the 1,257 basal genes identified, 277 (22%) were also identified in the stromal population. This suggests that the basal population expresses stromal genes, which may result from and assist with the communication between the two cell populations. This analysis demonstrates that our gene sets biologically validate with an independent enrichment of adult virgin cell subtypes and are conserved in human, confirming the identity of the enriched luminal and basal populations throughout the development of the mammary gland.

Based on the sorting conditions we used, the enriched basal epithelial population appears to be more transcriptionally dynamic than the luminal population. From the temporal analysis, we found that the majority of the cell population specific genes are temporally regulated during pregnancy, lactation and involution. These results suggest that the expression changes observed in the sorted populations reflect functional changes that occur during postnatal mammary gland development.

### Analysis of Lactation Specific Pathways Reveals Novel Functional Targets in Each Cell Population

To establish whether the transcriptome dynamics during pregnancy, lactation and involution reflect the developmental function of these cell subtypes, we tested for the enrichment of the time point and stage specific genes in functional groups annotated in the gene ontology (GO), KEGG and Reactome databases^52^,^53^,^54^. We set a threshold of FDR < 0.01 to determine the most statistically significant enrichment classifications. No significant KEGG pathways met this threshold; results from the GO analysis are available in Supplementary Table S3. We centered our results on the findings from the Reactome analysis because they contain well-curated pathways.

In the luminal population, we observed an upregulation of genes involved in lipid biosynthesis during lactation, specifically through SREBP (sterol regulatory element-binding protein) transcriptional regulation (Supplementary Figure S4 and Table S4). The Srebp family of transcription factors is important for milk lipid production across multiple species^55^,^56^,^57^. When we focused on the genes upregulated within this pathway, we uncovered known Srebf1 targets *Idi1*, *Mvd* and *Pmvk* as potential novel regulators of cholesterol biosynthesis during lactation specific to the luminal population (Supplementary Figure S4). These results support Srebf1 as a key transcription factor regulating the functional differentiation of luminal epithelial cells and identifies novel targets in the cholesterol biosynthesis pathway.

**Figure 3:**
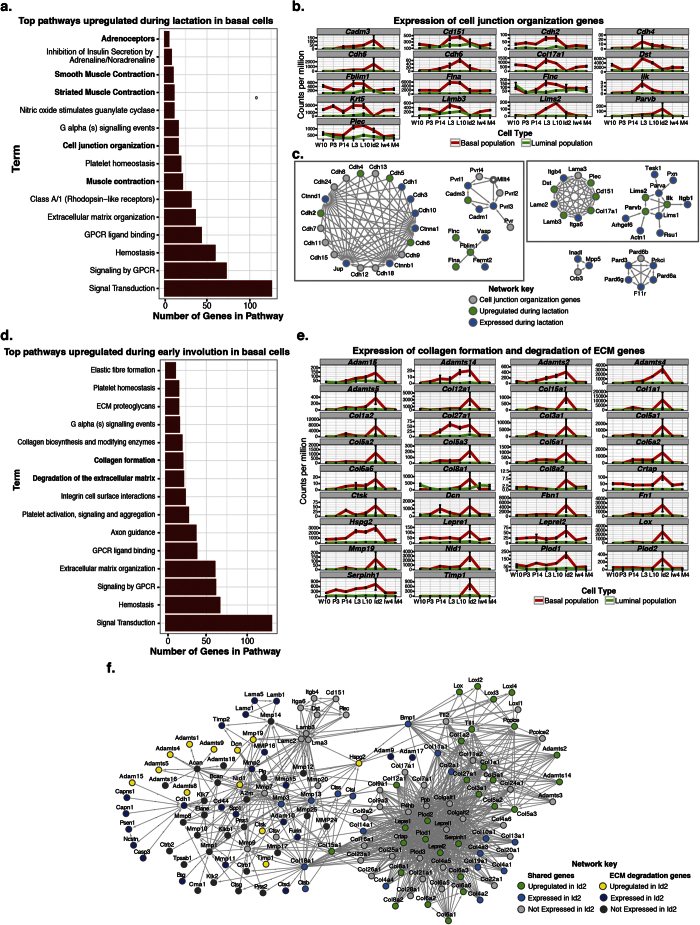
Cell-ECM interaction in basal cells during lactation and involution. (**a,d**) Top 15 Reactome pathways (FDR < 0.05) upregulated during lactation (**a**) and involution day 2 (**d**) in basal epithelial cells. Pathway terms are plotted on the y-axis and the total number of genes observed in each pathway is plotted on the x-axis. (**b,e**) Expression trajectory of identified cell junction organization genes (**b**, bold in **a**) and collagen formation and extracellular matrix degradation genes (**e**, bold in **d**) in basal (red line) and luminal (green line) populations. Normalized counts per million are plotted at each developmental time point. Error bars represent standard errors. (**c,f**) Functional Interaction (FI) network analysis of cell junction organization genes (**c**) and collagen formation and extracellular matrix degradation genes (**f**). Cell junction organization genes are subclassified by adherens junction interactions (dashed box), cell-extracellular matrix interaction (solid box) and tight junction interaction (no box) genes (**c**). Grey arrows (edges) represent direct interaction event. Grey and dark grey nodes are all genes in the pathway that are not expressed in the basal cells during lactation (**c**) and early involution (**f**). Green and yellow nodes are genes upregulated specifically during lactation (**c**) and early involution (**f**) in the basal population, while dark and light blue nodes represent genes whose expression is detected in the basal population during lactation (**c**) and early involution (**f**). All enriched reactome pathways are listed in Supplementary Table S4. See Supplementary Figure S4 for luminal reactome analysis results.

As expected, lactation in the basal epithelium is primarily characterized by the upregulation of muscle contractile genes (FDR = 1.25 × 10^−14^, Fig. 3a and Supplementary Table S4). We observed enrichment for adrenoceptors, specifically α1- and α2-adrenoceptors (*Adra1b*, *Adra1d*, *Adra2a*, *Adra2b* and *Adra2c*). Additionally, we found an enrichment of genes involved in Gα_q_ signaling events and PLCβ mediated events (FDR = 2.37 × 10^−3^ and 5.35 × 10^−4^, respectively Supplementary Table S4), primary downstream mediators of α1-adrenoceptor signaling^58^. Considering these findings and their established contractile function in smooth muscle cells^59^, we propose that α-adrenoceptors also play a role in regulating myoepithelial cell contraction. We also identified a novel, significant association with genes implicated in cell junction organization (Fig. 3a). The expression trajectories of the genes within this pathway demonstrated that they are specifically upregulated in the basal population during lactation (L3 and L10 time points, Fig. 3b). Functional interaction analysis revealed that several genes involved in adherens junction and cell-extracellular matrix (ECM) interactions (including type1 hemidesmosome assembly) were upregulated specifically during lactation in the basal cells (Fig. 3c, left and right boxes respectively). Adherens junction gene enrichment was centered on the identification of several cadherins (*Cdh2*, *Cdh4*, *Cdh5* and *Cdh6*), filamins (*Flna* and *Flnc*) and the filamin binding protein (*Fblim1*), which links the structural and motile functions of the actin cytoskeleton to cellular signaling^60^,^61^. The enrichment of genes involved in cell-ECM revolved around the identification of the lactation-specific upregulation of the ILK-PINCH-Parvin (IPP) complex (*Ilk*, *Lims2* and *Parvb*). IPP functions through its interaction with β1 integrin (*Itgb1*), also expressed in basal cells during lactation (Fig. 3c), and has been proposed to function as a bridge between cell adhesion and differentiation in the developing mammary gland^62^. Collectively, we identified novel receptors and cell-cell as well as cell-ECM interacting partners that are central to the function of basal cells during lactation.

### Luminal and Basal Epithelial Cells Have Independent Roles in Coordinating Tissue Remodeling During Early Involution

Early involution is characterized by an increased expression of genes involved in iron uptake, solute transport, by SLC (solute carrier) transporters, phagosomal maturation and endocytosis in the luminal cells (Supplementary Figure S4 and Table S4). The increase in iron uptake and solute transport supports previous associations between iron metabolism and apoptosis during mammary gland involution^63^,^64^. Lactoferrin (*Ltf*) binds and transports iron and functions in silencing casein milk proteins during early involution^65^. Consistent with previous reports, we found that this gene is also upregulated in the luminal cells during early involution (Supplementary Figure S2)^34^,^63^. The upregulation of phagosomal and endocytosis related genes support recent studies demonstrating that autophagic machinery proteins in mammary epithelial cells regulate phagocytic clearance of dead cells during involution^66^. We identified key vacuolar ATPase (V-ATPase) components, an enzyme located on the plasma membrane and important in phagocytosis, as being upregulated specifically during early involution in the luminal cells (Supplementary Figure S4). Furthermore, we demonstrated that other proteins within the complex, as well as members of the NADPH-oxidase enzyme complex, are expressed in luminal cells during early involution (Supplementary Figure S4). This data supports a role for luminal epithelial cells in the clearing of both nutrients and cells from the epithelial lumen.

Involution day 2 in the basal cells was largely characterized by the upregulation of tissue remodeling genes, such as those involved in platelet activation and aggregation, degradation of the extracellular matrix (ECM) and collagen formation (Fig. 3d). We observed a significant overlap in the degradation of the extracellular matrix and collagen formation genes, suggesting a coordinated role in the breakdown and reformation of the ECM during the second day of involution. Specifically, we identify cathepsin K (*Ctsk*), perlecan (*Hspg2*), procollagen C-endopeptidase enhancer (*Pcolce*) and tolloid-like1 (*Tll1*) as novel regulators of the tissue remodeling process expressed by the basal epithelium (Fig. 3e,f). Together, this data supports a model for the second phase of involution in which luminal cells regulate the clearance of dead cells and debris, while the basal cells regulate the physical process of tissue remodeling.

### Opposing Igf1r and Insr Function in Basal Cells

**Figure 4:**
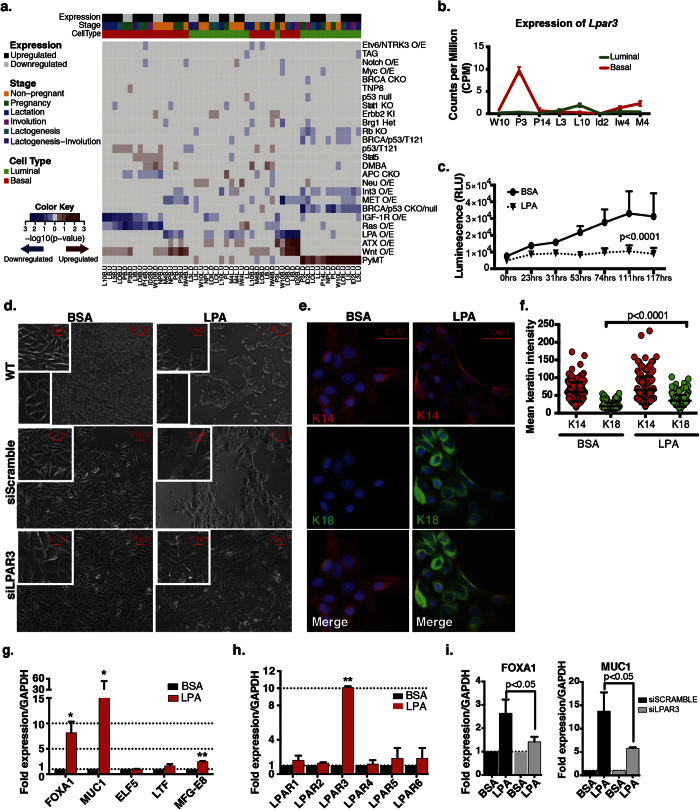
Lpar3 promotes LPA induced differentiation in basal mammary epithelial cells. (**a**) Heatmap of FWER p-values obtained from GSEA of the enrichment of the spatio-temporal gene sets (columns) obtained from various mouse models of mammary tumorigenesis (rows) in which genes were over expressed (O/E), conditionally knocked-out (CKO), knocked-out (KO) or knocked-in (KI) (red boxes represent association with upregulated genes, blue boxes associations with downregulated genes). (**b**) Expression trajectory of Lpar3 in the luminal and basal populations. Normalized counts per million are plotted at each developmental time point. Error bars represent standard errors. (**c**) Growth rate of HME50 cells in the presence of LPA (diamond dotted line) relative to mock treated (BSA) cells (circle continuous line) during 117 hrs of treatment. (**d**) Brightfield images of mock treated HME50 (left column) and LPA treated (right column). Cells were imaged cultured in growth medium (WT, top row), 48 hr post-transfection with a scrambled siRNA (middle row), or 48 hr post-transfection with a siRNA targeting LPAR3 (bottom row). Insert show enlarged representative areas of the main image. (**e**) Immunofluorescence of HME50 stained for cytokeratin 18 (green), cytokeratin 14 (red), and DAPI (blue) in the presence of mock treated controls or LPA. (**f**) Quantification of K14 and K18 signal intensity in mock controls versus LPA exposed HME50. (**g**) mRNA expression levels of luminal differentiation makers (*FOXA1* and *MUC1*) and milk production genes (*ELF5*, *LFT* and *MFG-E8*). Black columns represent mock treated HME50 cells and red columns LPA exposed cells (48 hr) (*p < 0.05). (**h**) mRNA expression levels of known LPAR receptors in HME50 cells upon 48 hr LPA (red columns) relative to mock treated cells (black columns) (**p < 0.01). (**i**) mRNA expression levels of *FOXA1* and *MUC1* in HME50 cells upon 48 hr LPA exposure after transfection with a scrambled siRNA (black columns) or a LPAR3 specific siRNA (grey columns). (**g–i**) Values are expressed as fold difference relative to GAPDH and normalized against the mock treated (BSA) controls. Error bars represent SD between three biological replicates, differences between groups were calculated using a t-test.

To further characterize our cell populations during development and identify potential population-specific gene expression changes that may be significant in tumorigenic processes we conducted a gene set enrichment analysis (GSEA) by comparing our spatio-temporal gene sets (separating the up- and down-regulated genes) to a published database comprising expression data from 24 different mouse models for breast cancer (Fig. 4a)^67^. When we set a cutoff of a FDR of 0.05, we found a significant enrichment of genes upregulated in the basal population during lactation and downregulated in the insulin-like growth factor 1 receptor (Igf1r) overexpression mouse model relative to the other tumor models. Igf1r has been shown to be necessary for normal alveolar proliferation and differentiation during pregnancy^68^. However, a basal cell specific function for Igf1r has yet to be established. We hypothesized that Igf1r signaling is diminished in the basal cells during lactation to inhibit proliferation and promote terminal cellular differentiation. To gain insight into a potential function of Igf1r in the basal epithelium during lactation, we observed the expression trajectories of Igf1r signaling associated genes and conducted a GO analysis on the identified gene targets. Our trajectory analysis demonstrates though that Igf1r was consistently expressed throughout development in both cell populations (Supplementary Figure S5). The basal specific expression of various insulin-like growth factor binding proteins (Igfbps 2, 4 and 6) and the inhibitor of Igf1r signaling, *Grb10*, suggested that these factors may restrict the signal transduction of Igf1r in the basal cells during lactation. GO analysis of the targets downregulated by Igf1r signaling (n = 339 L10, n = 422 lactation and n = 304 overlapping genes) and upregulated during lactation revealed that many of these genes are involved in development and differentiation processes (Supplementary Table S5). Analysis of the molecular functions of these genes based on their GO reveals that 26 of the 304 overlapping genes identified are classified as transcription factors including members of the Homeobox (Hox), T-box (Tbx), nuclear factor of activated T cells (Nfatc) and Kruppel-like factor (Klf) families of transcription factors (Supplementary Tables S6). Taken together, these data support a model in which Igf1r signaling promotes proliferation during earlier mammary development and this signaling is inhibited specifically in basal cells during lactation (possibly through Grb10 and Igfbp activity) to promote the expression of genes important for promoting cellular differentiation.

Additionally, we observed upregulation of *Insr* (insulin receptor), the insulin receptor substrates (*Irs1/2*) and *Igf2* during lactogenesis. Considering that Igf2 regulates alveologenesis and Igf2 binds to the A isoform of the Ir expressed in MECs^69^, we propose that local Igf2 promotes differentiation through Ir/Irs signaling^70^,^71^,^72^. Collectively, these findings support previous studies demonstrating that Igf1r promotes and regulates proliferation during early pregnancy, while Ir (insulin receptor) inhibits proliferation and promotes differentiation during lactogenesis^68^,^73^.

### ATX-LPA Axis is Activated in Basal Cells During Early Pregnancy and Involution

As the autotaxin–lysophosphatidic acid (ATX-LPA) signaling axis is one of the important survival factors and contributes to tumorigenesis, we investigated this further. We detected a significant correlation between the gene set downregulated in the basal cells during early involution and downregulated in the LPA receptor overexpression model (Fig. 4a). This suggests that LPA receptor signaling activation may take place during early involution. Given our initial discovery that GPCR signaling is important during lactogenesis and involution in the basal epithelial compartment and the fact that LPA receptors are GPCRs, we investigated their association with early involution further. To determine which receptor may be activated, we assessed the expression of the three LPA receptors overexpressed in the murine breast cancer model and *Enpp2* (ATX) at the gene expression and protein levels. We found that *Enpp2* is expressed specifically in the basal population with the highest induction during late pregnancy and into early involution (Supplementary Figure S6). Of the LPA receptors, *Lpar1* is uniquely upregulated during early involution in the basal population (Supplementary Figure S6). We confirmed this expression at the protein level and demonstrate that cells with high levels of cytokeratin 14 (K14^hi^) express Lpar1 at a higher level than K14 low expressing cells (K14^lo^) (Supplementary Figure S6). This suggests a potential function for Lpar1 during early involution.

The other LPA receptor expressed in the mammary epithelium, *Lpar3*, was specifically upregulated during early pregnancy in the basal epithelial population (Fig. 4b). Because of the specific timing of expression in basal cells, we investigated this gene further. To confirm the expression pattern at the protein level, we used confocal microscopy to test for Lpar3 expression in early pregnancy (P3.5) and week 10 (W10) mammary tissues. We observed low levels of Lpar3 (Lpar3^lo^) throughout the mammary epithelium (Supplementary Figure S7). However, a subset of cells demonstrated high levels of Lpar3 expression (Lpar3^hi^) irrespective of K14 levels, suggesting the presence of a LPA responsive subpopulation of cells (Supplementary Figure S7). There were no detectable Lpar3^hi^ or Lpar3^lo^ regions of interest (ROIs) in the non-primary antibody negative control, and we did not detect these staining patterns in the W10 tissue, supporting that the Lpar3 staining is accurately reflective of expression status (Supplementary Figure S7). We did not detect any *Lpar3* transcript in our enriched luminal population (Fig. 4b); therefore the Lpar3^hi^ cells sort with the basal population during early pregnancy.

To further characterize the basal cell specific activity of the LPA receptor, we exposed normal spontaneously immortalized human mammary basal cells (HME50)^74^ to LPA in culture for 24 to 72 hrs. LPA exposure in HME50 cells significantly delays growth (p = 8.4108 × 10^−8^) (Fig. 4c) and prompts extensive morphological changes in the 2D pattern of cell growth suggestive of *in vitro* differentiation that is evident as early as 24 hr (Fig. 4d, top row). Mock treated HME50 cells display the typical morphology of elongated myoepithelial cells, while LPA treated cells have a more cuboidal morphology characteristic of cells generating polarized epithelia. In addition, LPA exposure induces the formation of protrusion resembling lamellipodia, the cytoplasmic sheets that extended at the front of migrating cells (Fig. 4d, top row, bottom left inserts). Staining for luminal K18 and basal K14 specific markers show a significant increase in the expression of the luminal marker K18 in LPA exposed basal cells when compared to the mock treated control cells with some cells expressing both K18 and K14 markers suggesting the expression of markers for both epithelial cell lineages (Fig. 4e,f). To confirm that basal HME50 cells exposed to LPA undergo a differentiation switch, we measured the expression levels of two well characterized mammary epithelial luminal differentiation markers, Forkhead Box A1 (*FOXA1*) and Mucin1 (*MUC1*), as well as three genes known to be expressed in cells primed for milk production, E74 Like ETS Transcription Factor 5 (ELF5), Milk Fat Globule-EGF Factor 8 Protein (*MFG-E8*) and Lactotransferrin (*LTF*) (Fig. 4g). LPA exposure of HME50 cells for 48 hrs induces a significant up-regulation of both luminal differentiation markers tested (*FOXA1*, 8.1^+/−^ 2.2 fold, *MUC1* 16^+/−^ 5.3 fold); the milk production marker *MFG-E8* was also significantly up-regulated (2.4^+/−^ 0.1 fold) while *LFT* showed a trend for up-regulation but the difference did not reach statistical significance (1.6^+/−^ 0.34 fold). *ELF5* did not show differential expression (0.9^+/−^ 0.13 folds). Collectively, our data suggest that under these experimental conditions, LPA exposure reduces proliferation and prompts the differentiation of the basal population. As milk production genes were upregulated, these data support that LPA may contribute to the differentiation observed during pregnancy.

LPA is a small, ubiquitous phospholipid that acts as an extracellular signaling molecule by binding to and activating at least six known G protein-coupled receptors LPAR1 to LPAR6. All six LPAR genes are expressed in HME50 cells (Supplementary Figure S7) with *LPAR1*, *LPAR2* and *LPAR6* expressed at higher levels in non-LPA treated HME50 cells. Upon LPA exposure (48 hr) *LPAR3* alone becomes significantly up-regulated at the mRNA level relative to mock treated controls (p = 0.0083) (Fig. 4h). We next used small interfering RNA (siRNA) using a combined pool of four different individual siRNAs to silence the expression of *LPAR3* in HME50 basal cells. We confirmed that 48 hr post transfection *LPAR3* mRNA levels were down-regulated ~70% relative to mock treated HME50 cells (~30% after 24 hr, Supplementary Figure S7). Morphologically, *LPAR3* down-regulation impairs the LPA induced morphological changes observed in wild-type and mock transfected cells (Fig. 4d bottom row), suggesting that under our experimental conditions LPAR3 is a key mediator of the morphological differentiation switch induced by LPA. To confirm that LPAR3 indeed mediates the LPA induced differentiation in HME50 cells, we measured the levels of expression of the luminal markers *FOXA1* and *MUC1* as well as that of the milk production genes *ELF5*, *MFG-E8*, and *LTF* in siLPAR3 treated cells. We observed a significant reduction in the induction of mRNA levels for *FOXA1* and *MUC1* siLPAR3 treated relative to siScramble cells (*FOXA1* 1.4 versus 2.6 fold, and *MUC1* 5.7 versus 13.7 fold change relative to mock treated controls, p = 0.0303 and p = 0.045 respectively) (Fig. 4i). We also tested for a potential reduction in the mRNA levels of the milk production genes *ELF5*, *MFG-E8* and *LTF*, but we did not observe a significant inhibition in their LPA induced activation (data not shown).

**Figure 5:**
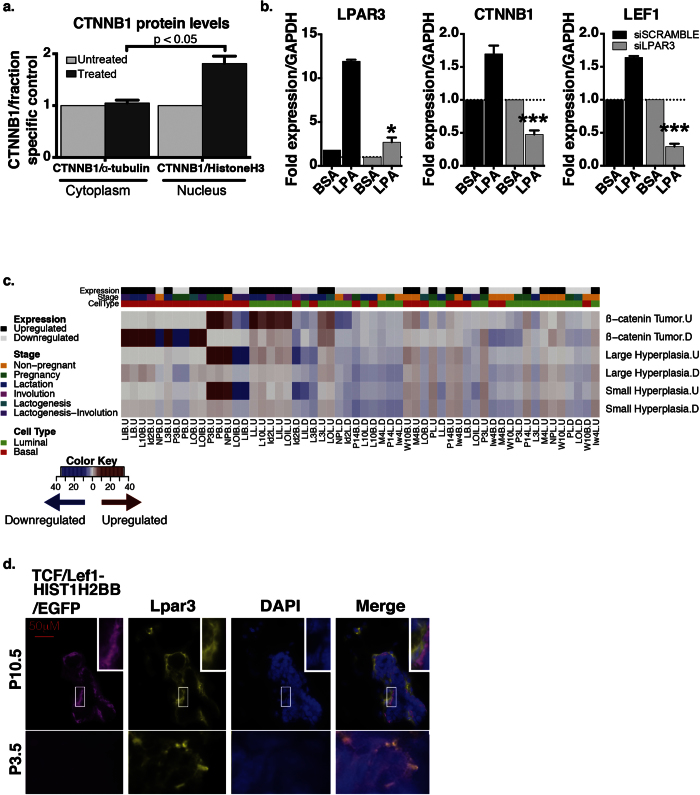
Lpar3 and β-catenin co-expression and activation during early pregnancy. (**a**) Quantification of β-catenin protein levels in cytoplasmic (left) and nuclear (right) fractions of LPA exposed HME50 cells versus controls. Error bars represent SD between three biological replicates, p = 0.031. (**b**) mRNA expression levels of *LPAR3*, *CTNNB1*and *LEF1* in HME50 upon 48 hr LPA exposure after transfection with a scrambled siRNA (black columns) or a *LPAR3* specific siRNA (grey columns). Values are expressed as fold difference relative to GAPDH and normalized against the mock treated controls. Error bars represent SD between three biological replicates, *p = 0.0049 and ***p = 0.003. (**c**) Heatmap representation of the enrichment of spatio-temporal gene sets (columns) in gene sets obtained from three stages of a mouse mammary tumor model in which β-catenin was overexpressed (GSE43825, rows). Each row represents genes upregulated (.U) and downregulated (.D) relative to normal samples. Dark red and blue blocks indicate a statistically significant enrichment of genes upregulated and downregulated in spatio-temporal gene sets, respectively (p < 0.01). (**d**) Fluorescent image of mammary gland alveoli at P10.5 and P3.5. Analysis performed on tissue sections of TcfLef-H2BGFP co stained for Lpar3 (yellow), and DAPI (blue).

Our breast cancer mouse model analysis revealed a slightly significant association between genes upregulated in P3.5 basal cells and with Wnt overexpression. We, therefore, hypothesized that this subpopulation of cells may have active β-catenin signaling, given that LPA receptors have been found to promote migration and differentiation through enhanced β-catenin signaling^75^,^76^,^77^. Additionally, our gene ontology analysis revealed a P3.5 basal cell-specific upregulation of Wnt signaling genes (Supplementary Table S3 and Figure S8). We therefore embarked on a dual *in vitro* and *in vivo* approach aimed to better characterize the interaction between LPA receptors and the β-catenin pathway in the mammary epithelium. To determine if LPA is sufficient to induce activation of β-catenin, we examined the nuclear localization of β-catenin in HME50 cells exposed to LPA after serum starvation. LPA exposure for 72 hours is sufficient to trigger translocation of β-catenin from the membrane compartment to the nucleus (Fig. 5a and Supplementary Figure S8).

We next questioned if LPAR3 activity directly mediates β-catenin activation. The protein results suggesting that β-catenin activation takes place in response to LPA exposure were confirmed at the mRNA level, as shown by a significant up-regulation of *LPAR3*, *CTNNB1* and *LEF1* in response to LPA (Supplementary Figure S8). However, both *CTNNB1* and *LEF1* mRNA up-regulation was abrogated in siLPAR3 treated cells (Fig. 5b). The trajectory analysis (Supplementary Figure S8) and *in vitro* results demonstrate that LPAR3 and β-catenin are co-expressed in mammary epithelial cells during early pregnancy and LPA exposure activates β-catenin in basal mammary epithelial cells through LPAR3 signaling. To further confirm that β-catenin signaling is active in the basal population during early pregnancy, we conducted an enrichment analysis of our gene sets in a dataset in which β-catenin was constitutively activated resulting in tumor formation (GSE43825). We found a significant enrichment (p < 2.64 × 10^−12^) of genes upregulated throughout pregnancy in the basal population in the genes upregulated due to β-catenin activation even in the earliest stage of tumor formation, small hyperplasia (Fig. 5c). The expression trajectories of these genes are depicted in Supplementary Figure S8. To assess β-catenin signaling activation during early pregnancy, we proceeded to analyze mammary glands isolated from TcfLef-H2BGFP transgenic mice at P3.5 and pregnancy day 10.5 (P10.5). The P10.5 time point was selected given that mRNA expression level of *Lef1* and *Tcf7* that peak at P14.5 and P3.5 in basal cells, respectively (Supplementary Figure S8). Glands from P10.5 mice, not P3.5, show the presence of clusters of cells within the basal compartment with GFP positive nuclei (Fig. 5d, magenta staining) indicative of active β-catenin signaling. GFP positive cells co-localize with Lpar3 positive cells at P10.5 as shown by immunofluorescence (Fig. 5d, merged layers) supporting our proposed model of Lpar3 basal specific activation via β-catenin during the early stages of pregnancy. Collectively, our results support the hypothesis that β-catenin is active during pregnancy in the basal population, and we propose that its activation through Lpar3 signaling events initiate a basal to luminal differentiation process that, during early pregnancy, is necessary to populate the mammary gland with cells of the milk production lineage.

## Discussion

The present work provides the first spatio-temporal transcriptome analysis of the luminal and basal population during mammary gland development. The ultimate goal of our analysis was to profile basal-specific regulatory networks. We present a technical and biological validation of the enrichment of luminal and basal mammary epithelial cells during mammary gland development. We demonstrate that Lin^−^CD24^+^ CD29^lo^ and Lin^−^CD24^+^ CD29^hi^ populations, luminal and basal epithelial cells respectively, consistently express their unique luminal and basal epithelial cell-specific markers throughout development. Finally, we show that the spatio-temporal control of key regulators of mammary gland development reflect their functional activity. The gene sets generated from these analyses have greatly improved our knowledge of regulators of epithelial cell activity throughout the various stages of mammary gland development. We identified over 4,000 basal specific temporally regulated genes, many of which define the identity and function of this largely unexplored cell population. Therefore, the present work provides an unprecedented dataset for the identification of new regulators of known pathways that can be further explored by functional characterization as we have performed here for the LPA signaling pathway.

We observed that the basal population has a more dynamic transcriptional profile than the luminal cells as reflected by the larger number of differentially expressed genes derived from the temporal analysis. We speculate that this could also be due, in part, to differences in population heterogeneity, with the basal population being more homogeneous than the luminal. The finding that the basal gene set is significantly more conserved than the luminal when comparing independently generated mouse and human gene sets supports this hypothesis^39^. The luminal population consists of a mosaic of hormonally responsive cell populations, while the basal population is thought to be composed primarily of myoepithelial cells with a minor stem/progenitor cell population. However, ductal and alveolar myoepithelial cells are structurally distinct and exhibit different expression patterns of smooth muscle cell markers, suggesting that a heterogeneous population of cells is also present within the basal layer^1^,^78^,^79^. Therefore, the data collected thus far do not allow for a definitive understanding as to whether basal epithelial cells are indeed more transcriptionally active, or if this is a reflection of the complexity and heterogeneity of these two main mammary epithelial cell subtypes. This data set, however, can be utilized to identify new markers for further enrichment of specific subpopulations of differentiated and progenitor mammary epithelial cells, making it possible to further explore the differences in transcriptional activity and functionality of these cells.

The transcriptional dynamics of the mammary epithelium throughout development are highly controlled by intrinsic and systemic signaling. Histologically, the basal epithelial cells create a barrier between the luminal epithelium and the surrounding stroma, and may serve as an intermediate of communication between both cellular compartments. During lactation, we observed an upregulation of genes involved in cell-ECM (ILK-PINCH-Parvin (IPP) complex and type I hemidesmosome components). Type I hemidesmosomes maintain the integrity and structure of tissues by linking the basal epithelial cell keratin cytoskeleton to the ECM, yet are able to quickly disassemble to allow for cell division, differentiation and migration^80^. Therefore, it is likely that these genes may be important in maintaining the structural integrity of the mammary epithelium during lactation where the epithelial compartment has drastically expanded and the myoepithelial cells are contracting to push milk through the ducts. Hormonal stimulation from prolactin and β1 integrin interaction with the ECM promotes milk protein production and epithelial cell differentiation through ILK signaling and activation of the Rho GTPase, Rac1^81^,^82^. Activation of Rac1 by ILK could occur through various ILK binding partners (Parvins, PINCH and Paxillin), however the mechanism by which ILK mediateds differentiation has yet to be determined^62^. We found that all genes in the IPP complex are expressed in basal cells during lactation, however we observe a basal population-specific upregulation of *Ilk*, *Lims2* (PINCH2), and *Parvb* (βParvin) suggesting that the ILK functions observed in previous studies are specific to the basal epithelium and occur through ILK/PINCH2 and ILK/βParvin interactions. Furthermore, evidence suggests that PINCH2 is not able to activate Rac1, however βParvin is a direct positive regulator of Rac1 activity^62^. We therefore propose that ILK/βParvin interactions promote Rac1 activity within the basal epithelium resulting in pro-differentiation signaling to the luminal epithelium, while the less understood ILK/PINCH2 interactions require further investigation.

Cell-cell adhesions through adherens junctions are largely mediated by cadherins, a family of proteins important for mediating cell-cell interactions and cell signaling. E- and P-cadherin stabilize the polarized cell shape of the epithelium and maintain the undifferentiated state of the mammary epithelium, respectively^2^,^3^,^83^,^84^. N-cadherin is upregulated in invasive tumors and promotes tumor cell metastasis, however little is known of its function in the normal mammary epithelium (reviewed in ref. 61). We found a basal population specific upregulation of *Cdh2* (N-cadherin) during lactation, supporting a lactation-specific function in this cell layer. Understanding the function of N-cadherin under physiological conditions in the lactating gland may help to better understand its role in tumorigenesis and the progression to metastasis.

We have evidence supporting basal-specific upregulation of α-adrenoceptors during lactation. Oxytocin, released from the pituitary gland, stimulates the contraction of myoepithelial cells by binding to the oxytocin receptor, a GPCR^85^,^86^. Adrenergic receptors (AR) are essential to cardiac physiology and the α_1_-AR subclass (α_1A_-, α_1B_- and α_1D_-AR) signal through coupling to G_q_/11 (Gα_q_) G-proteins and activate phospholipase Cβ1 (PLCβ1), which results in calcium release from intracellular stores and smooth muscle cell contraction^87^. Additionally, we demonstrated a significant enrichment of genes involved in Gα_q_ and PLCβ1 mediated signaling. Due to the well-characterized function of α_1_-ARs in regulating smooth muscle cell contraction, we propose that these receptors may also be responsible for simulating myoepithelial cell contraction. We also observed a basal cell-specific upregulation of α_2_-ARs. Given the cell subtype specific function of ARs and the known potential for AR subclasses to interact, further investigation into the function of these receptors in myoepithelial cells should incorporate both subtypes (reviewed in ref. 88).

Though we observed a basal cell specific association with Igf1r overexpression, all three receptors *Igf1r*, *Igf2r* and *Insr* were expressed in both cell subtypes. The expression patterns across development were relatively consistent when comparing both cell subtypes. This suggests that ligand accessibility and downstream mediators facilitate cell subtype-specific functions of these receptors. As previously reported, we showed that *Igf1* expression was increased in the luminal population during lactation and into early involution, both insulin receptor substrates (*Irs1/2*) increased expression during lactation, and the Igfbps were differentially expressed throughout the developmental time points exhibiting basal cell specificity (with the exception of *Igfbp5*). Basal cell specific upregulation of *Igf2* during lactation is consistent with its function as a mediator of morphogenesis during alveologenesis acting as a paracrine factor for the alveolar cells. However, we detected reduced levels of *Prlr* expression in the basal cells during lactation compared with non-pregnant time points (data not shown), suggesting that, in basal cells, *Igf2* expression may not be directly regulated by prolactin as described for mammary epithelial cells as a whole^70^,^71^. The upregulation of *Igf2r* during early involution supports a potential function in the turnover of Igf2 after lactation allowing for local *Igf1* mediated signaling during early involution. This turnover is important because, like Igf1, overexpression of Igf2 has been found to delay mammary involution by increasing cell survival^89^. Further functional studies are required to demonstrate these proposed functions *in vivo*.

Basal cell specific upregulation of *Igf1* during early involution suggests a potential role in involution. Previous studies have demonstrated that overexpression of Igf1 in the mammary gland results in enhanced focal cell survival during involution^90^,^91^ and that Igfbp5 interferes with Igf1 signaling, promoting apoptosis^92^. Our data suggests that the basal cells, through expression of *Igf1*, may be involved in orchestrating which cells survive, while luminal cell specific Igfbp5 induces apoptosis in the mammary epithelium during involution. Together, analysis of the spatio-temporal expression patterns of IGF signaling factors reveals novel insights into the coordinated signaling events during normal development, and more importantly highlights the importance of the basal epithelium in orchestrating development during lactation and post-lactational regression.

We obtained novel evidence that supports LPA receptor function in normal mammary gland development. A previous study has demonstrated that overexpression of LPA receptors is sufficient to promote mammary tumor formation and progression^93^, however their functions under physiological conditions have yet to be investigated. We demonstrate a basal cell specific upregulation of *Lpar1*, *Lpar3* and their upstream activator *Enpp2* (autotaxin). Considering that Lpar overexpression leads to a high frequency of chronic mastitis, which denotes chronic inflammation, we propose that Lpar1 expression promotes the early inflammatory response previously observed during early involution, which in turn also has the potential to induce the tumor-promoting environment during early involution^31^,^33^,^34^,^93^. We uncovered a novel role for Lpar3 in mediating the transition of basal epithelial cells into a more differentiated state committed to milk production. LPA, through its interaction with different high-affinity receptors, acts on different cell types and tissues to mediate a variety of cellular responses, including platelet aggregation, smooth muscle contraction, cell proliferation and differentiation, protection from apoptosis, stress fiber formation and tumor cell invasion^94^. In pancreatic cell lines, LPA was shown not to act as a mitogen, but as an efficacious stimulator of cell migration^95^. This is in agreement with our results where LPA hinder, proliferation in favor of differentiation. We also demonstrated that the expression profile of the basal population during pregnancy is significantly associated with active β-catenin signaling, an observation that is corroborated by the *in vivo* analysis of TcfLef-H2BGFP mice at P10.5. Given that a clear link between canonical Wnt signaling and the function of β-catenin in promoting lobulo-alveolar development during pregnancy has yet to be established (reviewed in ref. 96) and that Lpar3 has been found to activate β-catenin^77^, we now propose that signaling through Lpar3 may activate β-catenin in basal epithelial cells during early pregnancy. It is interesting to note that while at the mRNA level Enpp2 and Lpar3 increase in expression at early pregnancy, we do not observe active β-catenin signaling in the GFP reporter mouse until P10.5. While it is likely that the delay is caused by the time required for the establishment of the autocrine loop response, our results suggest that pregnancy could provide a robust model for the characterization of the activation of this specific pathway.

## Methods

### Mouse Maintenance and Mammary Gland Isolation

Four week-old male and female FVB/N (Taconic Laboratories Hudson, NY) were housed, bred and maintained under the approved animal protocol #20090502 (Institute for Animal Studies (IAS) of Albert Einstein College of Medicine). All methods were carried out in accordance with the guidelines approved by the Albert Einstein College of Medicine Institute for Animal Studies (IAS) (https://www.einstein.yu.edu/administration/animal-studies/). Animals were euthanized by an intraperitoneal injection with fresh 2.5% tribromoethanol (avertin) (Sigma Aldrich, St. Louis, MO) followed by cervical dislocation. Mice were mated at 8 weeks of age and the females were checked for plugs. A timeline of the sample collection strategy and the annotation used for the stages and time points are presented in the Supplementary Methods Table 1. Females with plugs were euthanized at 3.5 and 14.5 days post coitus (dpc). To further verify that the mice were pregnant, the uterus was checked for embryos. For the lactation time points, the mothers were euthanized at days 3 and 10 post-partum. Mice sacrificed for the involution time points were force weaned at 10 days post-partum. Involution day 2 mice were euthanized 2 days post forced weaning, and the involution week 4 (parous) mice were collected 28 days post weaning. The nulliparous (non-pregnant) age-matched controls were euthanized at 15 weeks (approximately 4 months old). Once euthanized, eight glands (second and third thoracic glands, the abdominal, and the inguinal glands) were collected from four mice and pooled for single cell suspension and FACS analysis (a total of 32 pooled glands for each sorting). The fourth mammary gland of a fifth mouse was fixed in 10% formalin and paraffin embedded for immunohistochemistry and immunofluorescence validation studies. The lymph nodes located in the abdominal and around the thoracic glands were removed before making the single cell suspension for cell sorting. To compensate for the variations in stages of the estrous cycle, mice were pooled into groups of four for sorting techniques.

TCF/Lef-H2BGFP mice were obtained by Jackson laboratory (Stock Number: 013752). Mice were bred in house and females with plugs were euthanized at 3.5 and 10.5 days post coitus (dpc). The third mammary gland isolated from a minimum of 3 mice for time point was fixed in cold acetone for 20 min and then embedded in Optimal Cutting Temperature (OCT) compound for sectioning.

### Single Mammary Epithelial Cell Suspension

Organoid isolation and single cell preparation were done as previously described^97^ with the following exceptions: phenol-red free DMEM/F12 was substituted for L15 medium (Invitrogen, Carlsbad, CA), tissue was finely minced manually on a glass petri dish on ice, enzymatic digestion was done in media consisting of phenol-red free DMEM/F12 supplemented with 2.5 μg/ml insulin in 0.01N HCl (Invitrogen, Carlsbad, CA), 2.5 μg/ml (Apo) transferrin in DMEM/F12 (MP Biomedicals, Santa Ana, CA), 10 ng/ml human EGF (Invitrogen, Carlsbad, CA), 0.5 μg/ml hydrocortisone (Sigma-Aldrich, St. Louis, MO), 50 μg/ml Gentamicin (Invitrogen, Carlsbad, CA) and 5% penicillin-streptomycin (Invitrogen, Carlsbad, CA) and combined with a 20X mixture of 300 U/ml collagenase A type 3 (Worthington, Lakewood, NJ) and 100 U/ml hyaluronidase (Sigma-Aldrich, St. Louis, MO). 20 μl 5 mg/ml DNaseI in 50% glycerol (Roche, Indianapolis, IN) was added to the mixture, glands were digested at 30 °C for 2 hours on a shaker at 200 rpm, and organoids were left at 4 °C overnight. Organoids were pelleted, and resuspended with 1 ml of pre-warmed 0.25% trypsin/EDTA (Invitrogen, Carlsbad, CA) containing 50 μg/ml freshly added DNaseI for (Roche, Indianapolis, IN) followed by digestion with trypsin for 2 minutes. To stop the digestion, 25 ml of wash media (DMEM/F12 with 5% FBS) was added to the tube, and the sample was pelleted at 800 rpm for 5 minutes. The dispase II digestion step was conducted by first adding 50 μg/ml DNaseI to 0.6 U/ml dispase II (Roche, Indianapolis, IN). The pellet was resuspended with the dispase solution and cells were digested at 37 °C for exactly 5 minutes. The cells were pelleted and the pellet was resuspended in ice-cold HBSS^cmf^. The cells were filtered over a 70 μm nylon filter (Thermo Fisher Scientific, Waltham, MA) and then over a 40 μm filter to remove any remaining clumps. The final single cell suspension was then pelleted, washed in PBS/EDTA, Dulbecco’s PBS^cmf^ (calcium-magnesium free) with 5 mM EDTA (Invitrogen, Carlsbad, CA).

### Fluorescence Activated Cell Sorting (FACS) Analysis

The isolated single cells were diluted to 10^7^ cells/ml in a FACS buffer, PBS^cmf^ supplemented with 2% BSA (bovine serum albumin) and 2% goat serum (Sigma-Aldrich, St. Louis, MO). 5 × 10^5^ cell aliquots were used as the negative and IgG controls, and the rest were stained with the antibody mix for positive and negative selection^28^ (see Supplementary Table S1). Staining was conducted by adding either the FACS buffer (negative control), fluorophore conjugated isotype mix (isotype control), or the fluorophore conjugated positive and negative selection cocktail to each well and mixing well by gently pipetting up and down. Cells were incubated protected from the light for 30 minutes on ice, then washed by adding a 50 μl FCS underlay. After pelleting, cells were resuspended in HBSS^cmf^ with 1% FCS to a final concentration of 10^6^ cells/ml. A compensation control was used to determine the true positive intensities for each fluorophore. FACS was performed as described elsewhere^28^,^43^,^97^,^98^ using the BD FACSAria II (BD Biosciences, San Jose, CA) equipped with a 100 μm nozzle and a pressure of 20 psi at 4 °C.

### RNA Amplification and Library Preparation for RNA-sequencing

We profiled a total of 47 samples for RNA-seq analysis, 3 biological replicates from each of the 2 cell subtypes across the 8 time points, with the exception of the lactation day 10 time point (only 2 biological replicates were assayed because of the low RNA yield). Due to the limited amount of RNA obtained from the sorted samples, the RNA was amplified using the Ovation RNA-Seq System V2 (NuGEN Technologies, San Carlos, CA). We followed the system protocol to amplify 5 ng of RNA for each sample, 100–200 pg of RNA was used as a low control to detect non-specific amplification. The resulting double-stranded cDNA (ds-cDNA) was stored at −20 °C for long-term storage. The Covaris-S2 Series ultrasonicator (Covaris, Woburn, MA) was used to fragment the ds-cDNA to around 300 bp. The libraries were made following the standard protocol for Illumina library preparation available online (http://www.einstein.yu.edu/docs/downloads/directional-transcriptome-seq.pdf). The protocol was followed with the exception of UNG treatment during the PCR amplification step. Samples were sequenced on the Illumina HiSeq (2000) Sequencing System (Illumina, San Diego, CA) by multiplexing six samples per lane across eight lanes. Normalized read counts and sequencing files in FASTQ format can be accessed at the Gene Expression Omnibus (GEO) repository under accession ID GSE88984.

### Statistics

All statistical methods, data analysis and enrichment analyses for the transcriptome and GEO data sets were conducted using the R statistical software^99^. To illustrate the expression differences for each gene we used the *ggplot2* package in R^100^ to plot the average of the normalized counts per million (CPM) across biological replicates and the standard error for each condition (conditions are defined in Supplementary Methods Table 1). Heatmaps were created using the heatmap.3 function in the GMD package^101^. The p-values for enrichment analyses were calculated using a hypergeometric distribution test using the R *stats package*^99^. For the functional experiments shown in Figs 4 and 5 statistical differences between BSA treated and LPA treated cells were calculated using a paired t-test. Differences between groups (Fig. 4c,f) were assessed using 2-way ANOVA (GraphPad Prism).

### Cell Culture and siRNA

Human Basal Myoepithelial Cells HME50 (gift of Professor Lindsay Hinck, Santa Cruz, University of California, USA) were grown at 37 °C with 5% CO_2_ in DMEM-F12 medium supplemented with 1% mammary epithelial cell growth supplement (MEGS), Cascade Biologics and 1% Penicillin-Streptomycin, GIBCO. For experimental purposes the cells were grown up to 50–60% confluency, trypsinized, counted with hemocytometer (Improved Neubauer) and used for further analysis. Serum starved HME50 cells were induced with 10 μM Lysophosphatidic acid (LPA, Avanti Polar Lipids, Alabama, USA) dissolved in PBS and 0.1% fat free BSA and added to the culture media. As a control, cells were suspended in DMEM-F12 medium with 0.1% fat free BSA only. The cells were incubated for 24 to 72 h after which they were either fixed for immunofluorescence, transfected for LPAR3 downregulation, or trypsinized and harvested by centrifugation at 1200 rpm for 10 minutes. The pellet was used for cell fractionation.

For silencing of LPAR3 we used the ON-TARGETplus LPAR3 siRNA (Dharmacon, Lafayette USA) and compared the results with HME50 cells treated using the Non-Targeting siRNA Control Pools. Si oligoes were resuspended using manufacture procedures in RNase-free 1x siRNA Buffer at a final concentration of 20 mM was delivered to HME50 cells using the Amaxa Nucleofector Technology (Program Y01). After transfections cells were allowed to recover for 24 hours after which the media was supplemented with LPA or vehicle.

To measure the growth rate response to LPA, serum starved HME50 cells plated in an optical white 96 well plate (Thermofisher, Waltham MA) were exposed to 10 μM LPA or vehicle (0.1% fat free BSA only) for 10 hrs after which the number of live cells was measured using the RealTime-Glo MT Cell Viability Assay (Promega, Madison WI) following manufacture procedures. Bioluminescent intensity was measured using a BioTek Synergy 4 Hybrid Microplate Reader (BioTek, Winooski VT).

## Additional Information

**How to cite this article**: Acosta, D. *et al.* LPA receptor activity is basal specific and coincident with early pregnancy and involution during mammary gland postnatal development. *Sci. Rep.* **6**, 35810; doi: 10.1038/srep35810 (2016).

**Publisher’s note**: Springer Nature remains neutral with regard to jurisdictional claims in published maps and institutional affiliations.

## Supplementary Material

Supplementary Information

Supplementary Table 2

Supplementary Table 3

Supplementary Table 4

Supplementary Table 5

Supplementary Table 6
