# Supplementary material for: LPA receptor activity is basal specific and coincident with early pregnancy and involution during mammary gland postnatal development
Source: Sci Rep. 2016 Nov 3;6:35810. doi: 10.1038/srep35810 (PMC5093903; doi:10.1038/srep35810)
Supplement: Supplementary Information [file srep35810-s1.pdf]

LPA receptor activity is basal specific and coincident with early pregnancy and involution during mammary gland postnatal development

Deanna Acosta[1], Susmita Bagchi[1], Pilib Ó Broin[1], Daniel Hollern[3], Silvia E. Racedo[1], Bernice Morrow[1], Rani S. Sellers[2], John M Greally[1], Aaron Golden[1], Eran Andrechek[3], Teresa Wood[4], Cristina Montagna[1,2]

[1] Department of Genetics, [2]Department of Pathology Albert Einstein College of Medicine, Bronx, NY 10461, USA
[3]Department of Physiology, Michigan State University, East Lansing, MI 48824, USA
[4]Department of Neurology and Neuroscience, Rutgers New Jersey Medical School, Newark, NJ 07101, USA

Supplementary Information

Figure S1

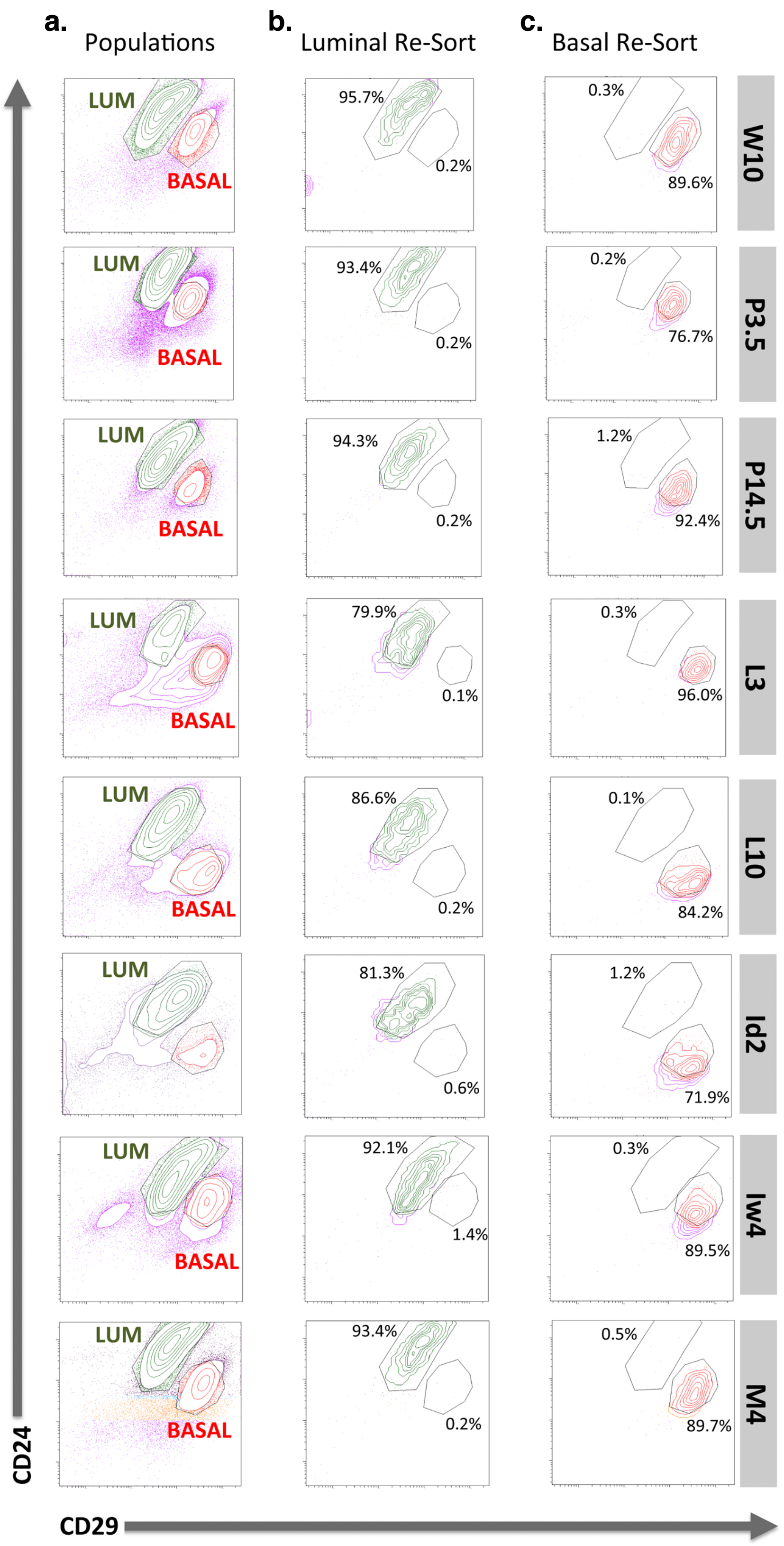
a.
Populations
b.
Luminal Re-Sort
c.
Basal Re-Sort
LUM
BASAL
95.7%
0.2%
0.3%
89.6%
W10
93.4%
0.2%
0.2%
76.7%
P3.5
94.3%
0.2%
1.2%
92.4%
P14.5
79.9%
0.1%
0.3%
96.0%
L3
86.6%
0.2%
0.1%
84.2%
L10
81.3%
0.6%
1.2%
71.9%
Id2
92.1%
1.4%
0.3%
89.5%
Iw4
93.4%
0.2%
0.5%
89.7%
M4
CD24
CD29

Figure S1. FACS enrichment of luminal and basal epithelial populations. (a) FACS contour plots of one representative sample per time point depicting the intensity of the CD24 and CD29 markers in the lineage negative population (purple points). Lin-CD24+CD29lo (luminal, LUM) and Lin-CD24+CD29hi (basal) populations are colored green and red, respectively. Each point in the plot represents a different cell. **(b** and **c)** Re-sort of 1,000 enriched luminal and basal epithelial cells, respectively, from each time point. The percentage of cells recovered in each population after the re-sort is noted next to its respective population on the graph. Each row represents a different time point as labeled in the grey boxes to the right of the graphs.

Figure S2

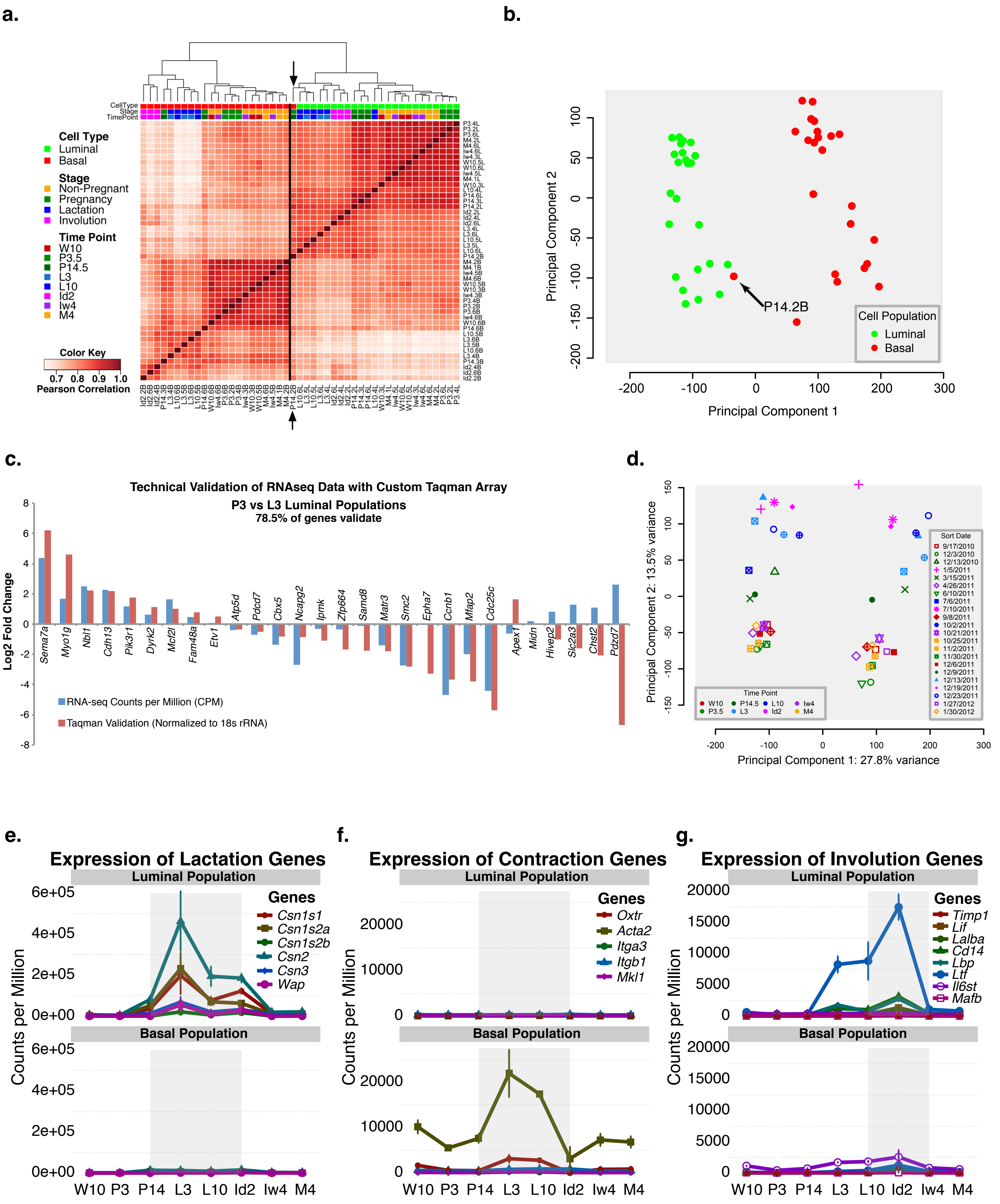

a.
Cell Type
Luminal
Basal
Stage
Non-Pregnant
Pregnancy
Lactation
Involution
Time Point
W10
P3.5
P14.5
L3
L10
Id2
Iw4
M4
Color Key
Pearson Correlation
b.
P14.2B
Principal Component 1
Principal Component 2
Cell Population
Luminal
Basal
c.
Technical Validation of RNAseq Data with Custom Taqman Array
P3 vs L3 Luminal Populations
78.5% of genes validate
Log2 Fold Change
RNA-seq Counts per Million (CPM)
Taqman Validation (Normalized to 18s rRNA)
d.
Principal Component 1: 27.8% variance
Principal Component 2: 13.5% variance
Sort Date
Time Point
e.
Expression of Lactation Genes
Luminal Population
Basal Population
Counts per Million
Genes
Csn1s1
Csn1s2a
Csn1s2b
Csn2
Csn3
Wap
f.
Expression of Contraction Genes
Oxtr
Acta2
Itga3
Itgb1
Mkl1
g.
Expression of Involution Genes
Timp1
Lif
Lalba
Cd14
Lbp
Ltf
Il6st
Mafb
W10 P3 P14 L3 L10 Id2 Iw4 M4

Figure S2. Identification of sample outliers and validation of RNAseq data. (a) Heatmap of the correlation matrix calculated across all samples using the Pearson method. Correlation scale ranges from blue to red, with red showing the highest correlation (R2 = 1). Each column is color-coded based on the biological covariates: cell type, stage and time point. Cell types are depicted in green (luminal cells) and red (basal cells). The stages are colored orange (virgin or non-pregnant), green (pregnancy), blue (lactation), and light purple (involution). The time points are highlighted in red (W10), light green (P3), green (P14), light blue (L3), blue (L10), light purple (Id2), purple (Iw4) and orange (M4). The black arrow marks the sample outlier. **(b)** Plot of the first two principal components from the principal component analysis (PCA) of all samples colored based on the cell type of the sample, luminal (green) and basal (red). The black arrow points to the sample outlier. **(c)** Log2 fold changes (P3 vs L3) in RNAseq (normalized counts per million, blue) and Taqman (normalized to 18S rRNA, red) analyses plotted for each gene (x-axis). **(d)** Plot of the first two principal components from the principal component analysis (PCA) of all samples colored based on the FACS sorting date of the sample and time points. **(e-g)** Expression trajectories of lactation **(e),** contraction **(f)** and involution **(g)** specific genes in the luminal (top) and basal (bottom) populations. Normalized counts per million are plotted at each developmental time point. Error bars represent standard errors.

Figure S3

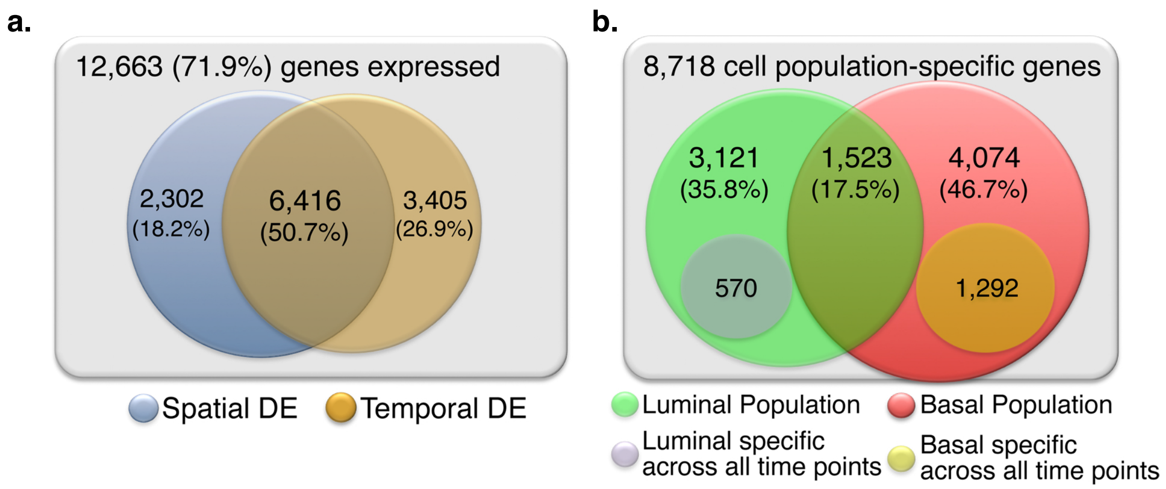
a.
12,663 (71.9%) genes expressed
2,302
(18.2%)
6,416
(50.7%)
3,405
(26.9%)
Spatial DE
Temporal DE
b.
8,718 cell population-specific genes
3,121
(35.8%)
1,523
(17.5%)
4,074
(46.7%)
570
1,292
Luminal Population
Basal Population
Luminal specific across all time points
Basal specific across all time points

Figure S3. Spatio-temporal analysis of differentially expressed genes, biological validation and species conservation of cell subtype-specific genes. (a) Venn diagram representing the number of spatially (blue) and temporally (yellow) differentially expressed genes. The total number of expressed genes is on the top left. The overlapping area represents genes that are both spatially and temporally differentially expressed. **(b)** Venn diagram representing the number of genes that are luminal (green) or basal (red) in at least one time point. The total number of cell population specific genes in at least one time point is on the top left. Overlapping area shows the number of genes that are both luminal and basal specific at different time points. Smaller circles represent the number of luminal specific (purple) and basal specific (yellow) genes across all 8 developmental time points. **(a** and **b)** The arbitrary threshold for determining the number of significant differentially expressed genes was a relatively stringent fold change of 2 and FDR of 0.01.

Figure S4

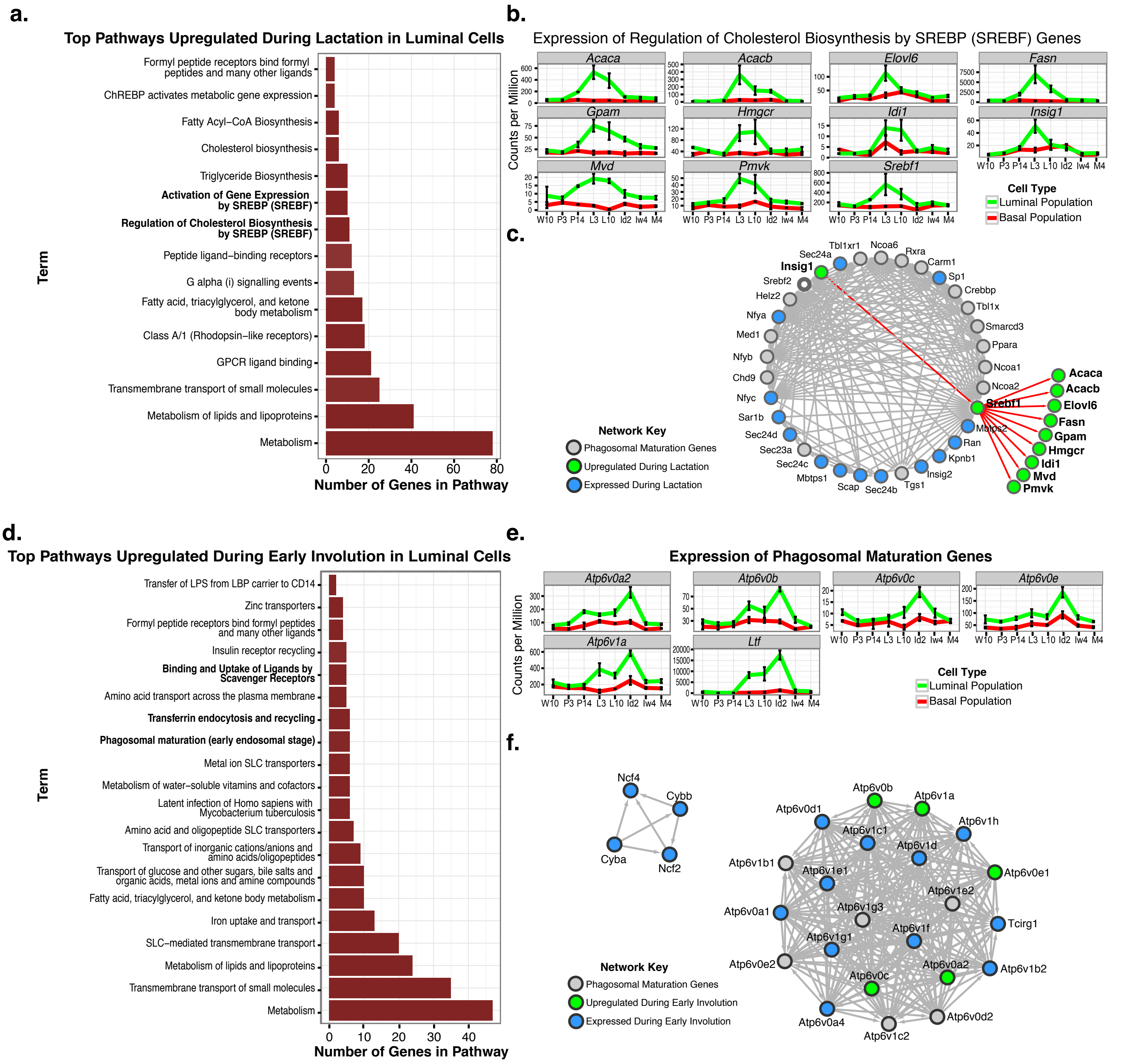
a.
Top Pathways Upregulated During Lactation in Luminal Cells
Formyl peptide receptors bind formyl peptides and many other ligands
ChREBP activates metabolic gene expression
Fatty Acyl−CoA Biosynthesis
Cholesterol biosynthesis
Triglyceride Biosynthesis
Activation of Gene Expression by SREBP (SREBF)
Regulation of Cholesterol Biosynthesis by SREBP (SREBF)
Peptide ligand−binding receptors
G alpha (i) signalling events
Fatty acid, triacylglycerol, and ketone body metabolism
Class A/1 (Rhodopsin−like receptors)
GPCR ligand binding
Transmembrane transport of small molecules
Metabolism of lipids and lipoproteins
Metabolism
Term
0 20 40 60 80
Number of Genes in Pathway
b.
Expression of Regulation of Cholesterol Biosynthesis by SREBP (SREBF) Genes
Acaca
Acacb
Elovl6
Fasn
Gpam
Hmgcr
Idi1
Insig1
Mvd
Pmvk
Srebf1
Counts per Million
W10 P3 P14 L3 L10 Id2 Iw4 M4
Cell Type
Luminal Population
Basal Population
c.
Network Key
Phagosomal Maturation Genes
Upregulated During Lactation
Expressed During Lactation
Insig1
Sec24a
Tbl1xr1
Ncoa6
Rxra
Carm1
Sp1
Crebbp
Tbl1x
Smarcd3
Ppara
Ncoa1
Ncoa2
Srebf1
Mbtps2
Ran
Kpnb1
Insig2
Tgs1
Sec24b
Scap
Mbtps1
Sec24c
Sec23a
Sec24d
Sar1b
Nfyc
Chd9
Nfyb
Med1
Nfya
Helz2
Srebf2
Acaca
Acacb
Elovl6
Fasn
Gpam
Hmgcr
Idi1
Mvd
Pmvk
d.
Top Pathways Upregulated During Early Involution in Luminal Cells
Transfer of LPS from LBP carrier to CD14
Zinc transporters
Formyl peptide receptors bind formyl peptides and many other ligands
Insulin receptor recycling
Binding and Uptake of Ligands by Scavenger Receptors
Amino acid transport across the plasma membrane
Transferrin endocytosis and recycling
Phagosomal maturation (early endosomal stage)
Metal ion SLC transporters
Metabolism of water−soluble vitamins and cofactors
Latent infection of Homo sapiens with Mycobacterium tuberculosis
Amino acid and oligopeptide SLC transporters
Transport of inorganic cations/anions and amino acids/oligopeptides
Transport of glucose and other sugars, bile salts and organic acids, metal ions and amine compounds
Fatty acid, triacylglycerol, and ketone body metabolism
Iron uptake and transport
SLC−mediated transmembrane transport
Metabolism of lipids and lipoproteins
Transmembrane transport of small molecules
Metabolism
Term
0 10 20 30 40
Number of Genes in Pathway
e.
Expression of Phagosomal Maturation Genes
Atp6v0a2
Atp6v0b
Atp6v0c
Atp6v0e
Atp6v1a
Ltf
Counts per Million
W10 P3 P14 L3 L10 Id2 Iw4 M4
Cell Type
Luminal Population
Basal Population
f.
Ncf4
Cybb
Cyba
Ncf2
Network Key
Phagosomal Maturation Genes
Upregulated During Early Involution
Expressed During Early Involution
Atp6v0b
Atp6v1a
Atp6v0d1
Atp6v1c1
Atp6v1h
Atp6v1d
Atp6v1b1
Atp6v1e1
Atp6v0e1
Atp6v1e2
Atp6v0a1
Atp6v1g3
Tcirg1
Atp6v1f
Atp6v1g1
Atp6v0e2
Atp6v0a2
Atp6v1b2
Atp6v0c
Atp6v0a4
Atp6v0d2
Atp6v1c2

Figure S4. Identification of novel SREBP target genes during lactation and core components involved in phagosomal maturation during Id2 in enriched luminal cells. (a and **d)** Top Reactome pathways (FDR < 0.05) upregulated during lactation **(a)** and involution day 2 **(d)** in luminal epithelial cells. Pathway terms are plotted on the y-axis and the total number of genes observed in each pathway is plotted on the x-axis. **(b** and **e)** Expression trajectory of identified regulation of cholesterol biosynthesis by SREBP genes **(b,** bold in **a)** and phagosomal maturation genes **(e,** bold in **d)** in basal (red line) and luminal (green line) populations. Normalized counts per million are plotted at each developmental time point. Error bars represent standard errors. **(c** and **f)** Functional Interaction (FI) network analysis of regulation of cholesterol biosynthesis by SREBP and phagosomal maturation genes **(c** and **f**, respectively**)**. Grey arrows (edges) represent direct interaction event. Grey nodes are all genes in the pathway not expressed in the luminal cells during lactation **(c)** and early involution **(f)**. Green nodes are genes upregulated specifically during lactation **(c)** and early involution **(f)** in the luminal population, while light blue nodes represent genes whose expression is detected in the luminal population during lactation **(c)** and early involution **(f)**.

Figure S5

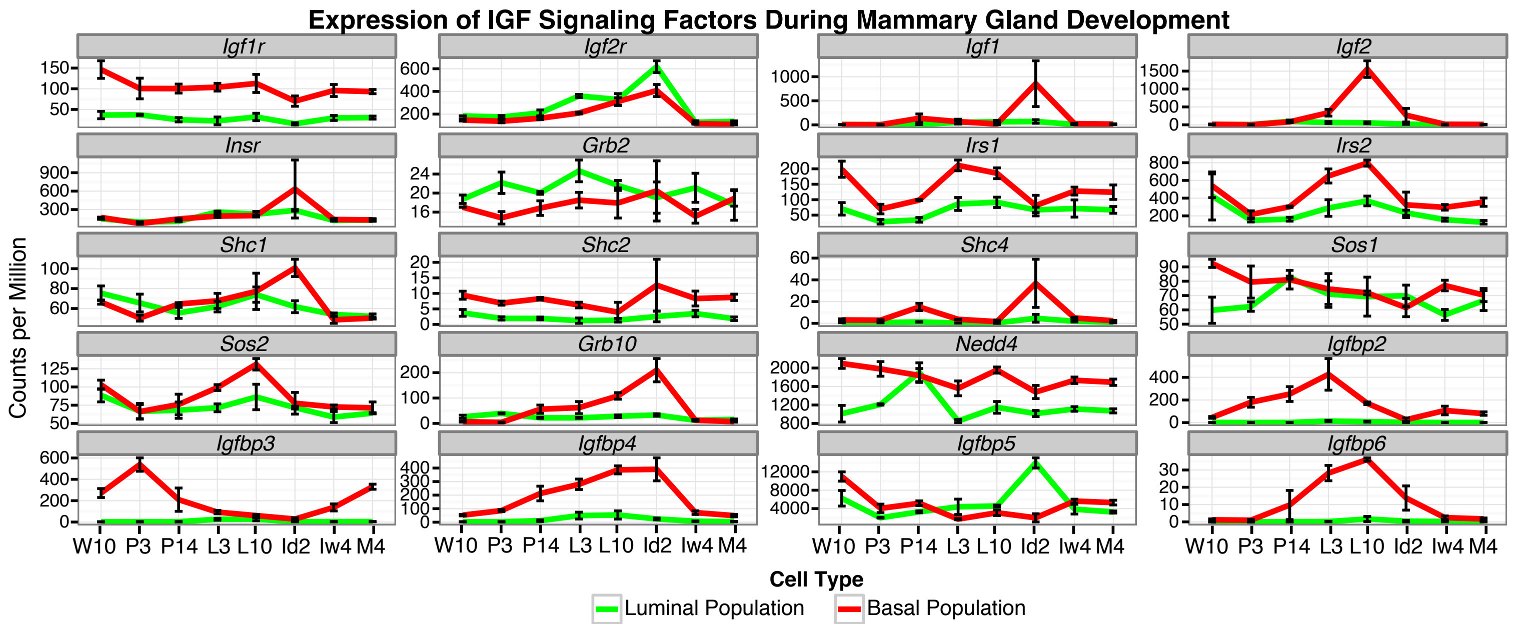

Expression of IGF Signaling Factors During Mammary Gland Development
Igf1r
Igf2r
Igf1
Igf2
Insr
Grb2
Irs1
Irs2
Shc1
Shc2
Shc4
Sos1
Sos2
Grb10
Nedd4
Igfbp2
Igfbp3
Igfbp4
Igfbp5
Igfbp6
Counts per Million
W10 P3 P14 L3 L10 Id2 Iw4 M4
Cell Type
Luminal Population
Basal Population

Figure S5. Expression of IGF signaling factors during development. Expression trajectory of identified IGF signaling factors in basal (red line) and luminal (green line) populations. Normalized counts per million are plotted at each developmental time point. Error bars represent standard errors.

Figure S6

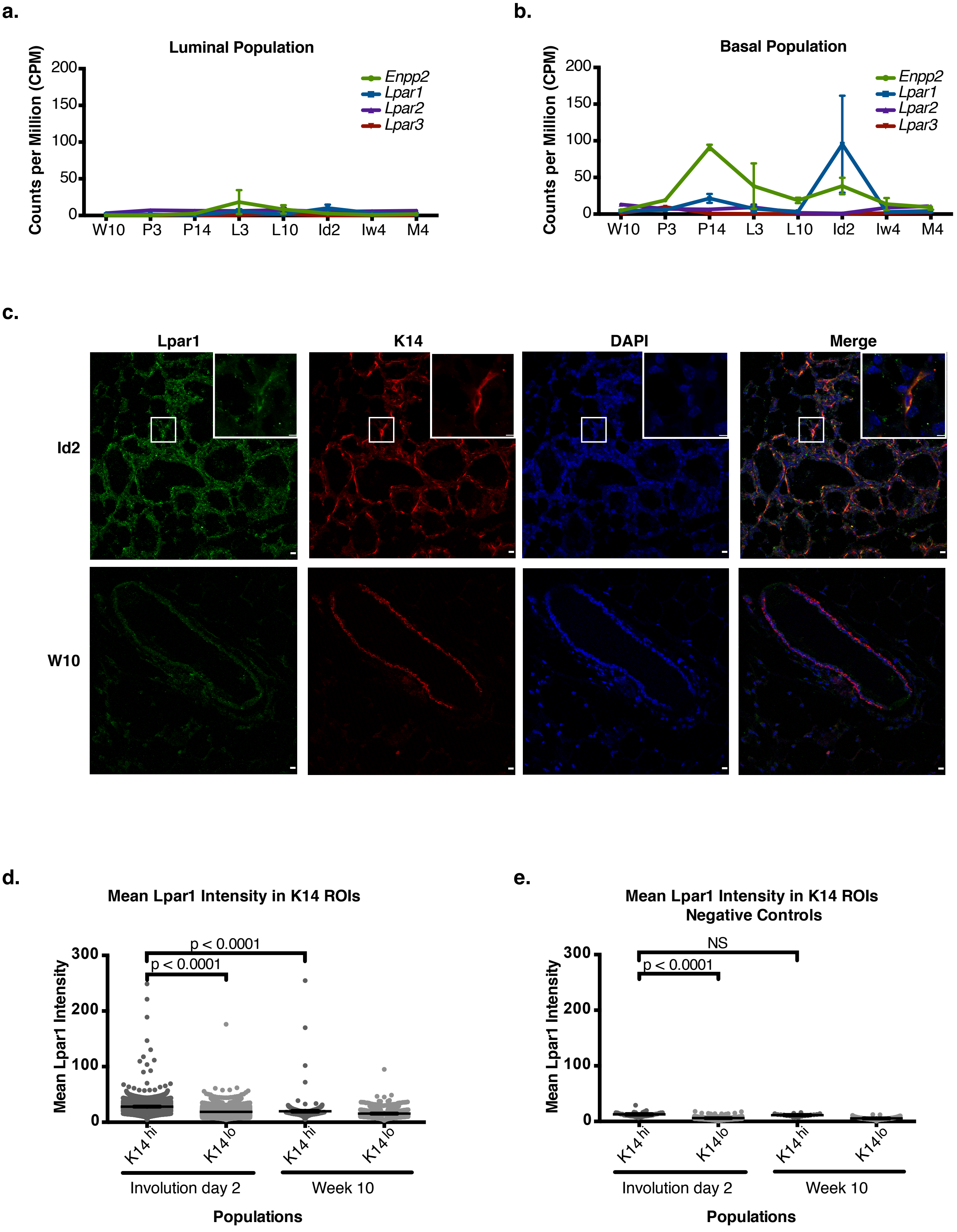
a.
Luminal Population
Counts per Million (CPM)
200
150
100
50
0
W10
P3
P14
L3
L10
Id2
Iw4
M4
Enpp2
Lpar1
Lpar2
Lpar3
b.
Basal Population
Counts per Million (CPM)
200
150
100
50
0
W10
P3
P14
L3
L10
Id2
Iw4
M4
Enpp2
Lpar1
Lpar2
Lpar3
c.
Lpar1
K14
DAPI
Merge
Id2
W10
d.
Mean Lpar1 Intensity in K14 ROIs
Mean Lpar1 Intensity
300
200
100
0
p < 0.0001
p < 0.0001
K14 hi
K14 lo
K14 hi
K14 lo
Involution day 2
Week 10
Populations
e.
Mean Lpar1 Intensity in K14 ROIs
Negative Controls
Mean Lpar1 Intensity
300
200
100
0
NS
p < 0.0001
K14 hi
K14 lo
K14 hi
K14 lo
Involution day 2
Week 10
Populations

Figure S6. Activation of Lpar1 in basal epithelial cells during early involution. (a and **b)** Expression trajectories of ATX-LPA axis genes in the luminal **(a)** and basal **(b)** populations. Normalized counts per million are plotted at each developmental time point. Error bars represent standard errors. **(c)** Laser confocal microscopy images of involution day 2 (Id2) and week 10 virgin (W10) mouse mammary tissue sections stained for Lpar1 (green), cytokeratin 14 (K14, red) and DAPI (blue). Images were taken at 40X magnification, scale bars represent 10μm for main panel images and 5μm for insets. **(d** and **e)** Plots of the mean intensity of Lpar1 staining in K14 regions of interest (ROIs) in tissue stained with **(d)** and without **(e)** primary antibodies. Analysis performed on one tissue section from four mice at each time point and staining condition.

Figure S7

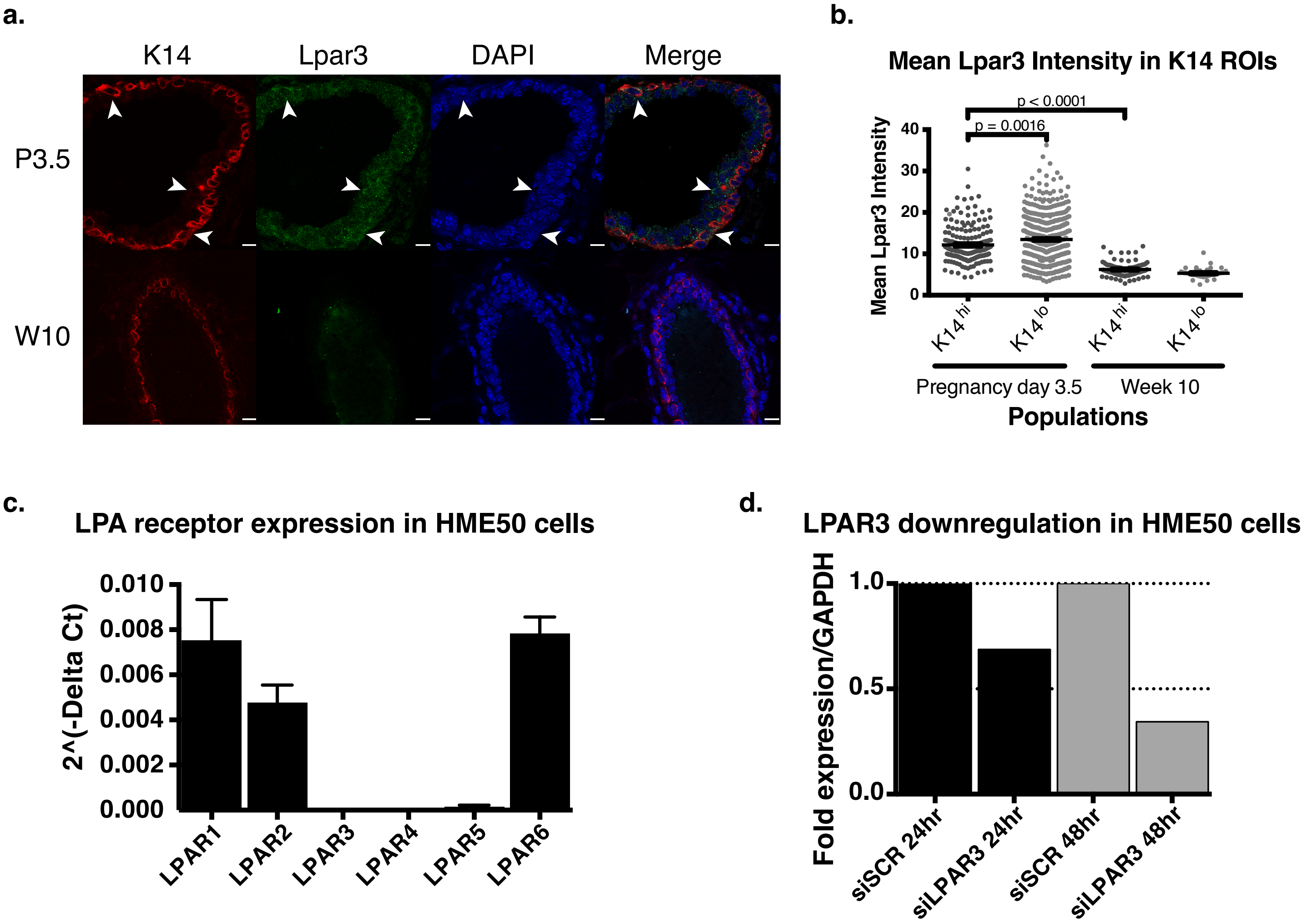

a.
K14
Lpar3
DAPI
Merge
P3.5
W10
b.
Mean Lpar3 Intensity in K14 ROIs
p < 0.0001
p = 0.0016
Mean Lpar3 Intensity
40
30
20
10
0
K14 hi
K14 lo
K14 hi
K14 lo
Pregnancy day 3.5
Week 10
Populations
c.
LPA receptor expression in HME50 cells
2^(-Delta Ct)
0.010
0.008
0.006
0.004
0.002
0.000
LPAR1
LPAR2
LPAR3
LPAR4
LPAR5
LPAR6
d.
LPAR3 downregulation in HME50 cells
Fold expression/GAPDH
1.0
0.5
0.0
siSCR 24hr
siLPAR3 24hr
siSCR 48hr
siLPAR3 48hr

Figure S7. β-catenin signaling is active during early pregnancy in the basal epithelial population. **(a)** Laser confocal microscopy images of pregnancy day 3.5 (P3.5) and week 10 virgin (W10) mouse mammary tissue sections stained for cytokeratin 14 (K14, red), Lpar3 (green), and DAPI (blue). Images were acquired at 63X. The scale bars represent 10μm. Arrowheads pinpoint to regions of LPA responsive subpopulation of cells. **(b)** Plot of the mean intensity of Lpar3 staining in K14 ROIs. Analysis performed on two tissue sections from one mouse at each time point. **(c)** mRNA expression levels of LPAR receptors in untreated HME50 cells. Error bars represent SD over three biological replicates. **(d)** mRNA expression levels of LPAR3 in HME50 cells 24 or 48 hrs post transfection with a scramble siRNA (black columns) or transfected with an interference RNA against LPAR3 (grey columns).

Figure S8

a.

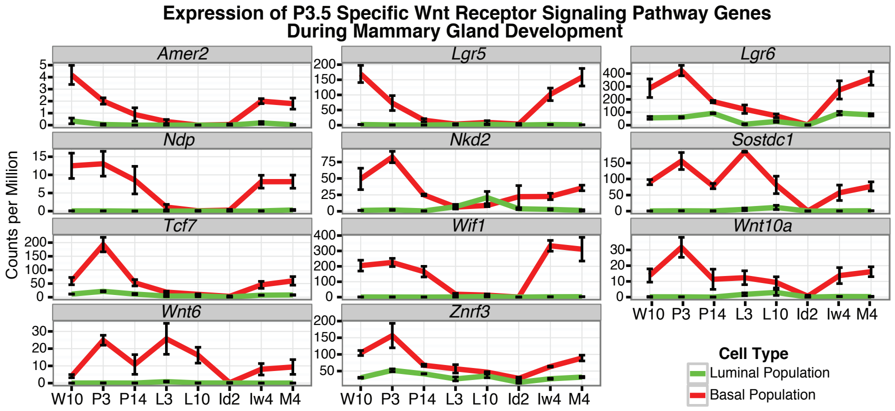

b.

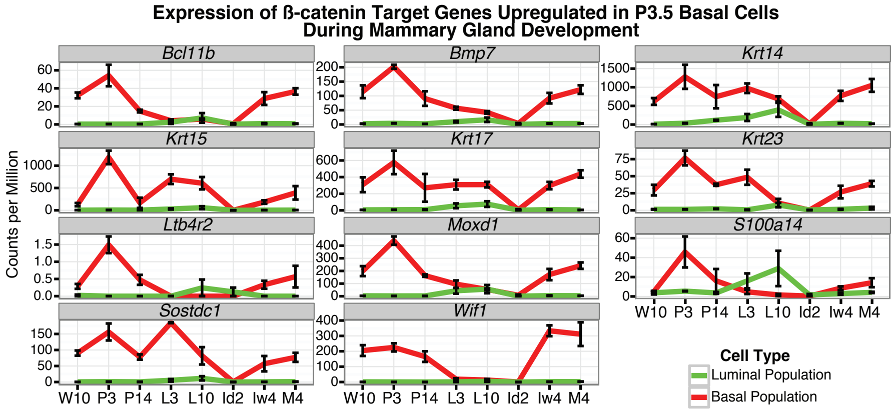

c.

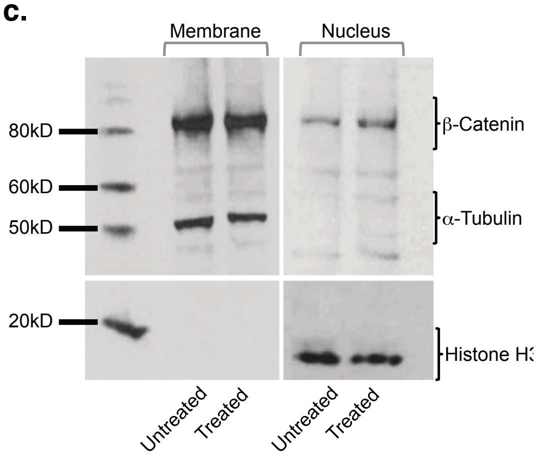

d.

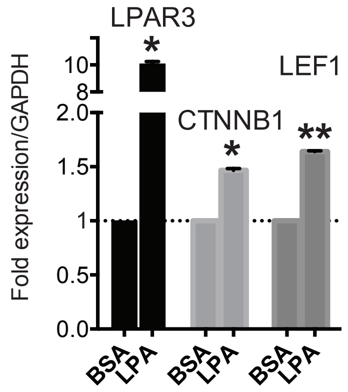

e.

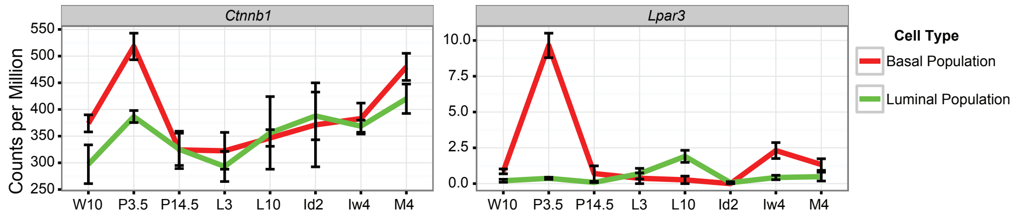

f.

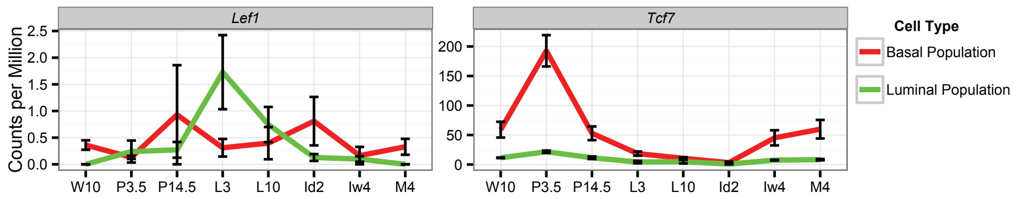

Figure S8. LPA activates β-catenin signaling in basal cells. (a and **b)** Expression trajectories of **(a)** Wnt receptor signaling pathway genes and **(b)** β-catenin target genes in luminal (green) and basal (red) epithelial cells throughout development. Normalized counts per million are plotted at each developmental time point. Error bars represent standard errors. **(c)** Western blot depicting bands corresponding to the levels of protein expression of β-Catenin (top, ~ 80kD band) and α-tubulin (top, ~50kD band) in membrane and nuclear fractions of HME50 mock treated or LPA treated HME50 cells. The bottom panel shows the expression levels of the nuclear control protein Histone H3 in all cellular extracts. **(d)** mRNA expression levels of LPAR3, CTNNB1, and LEF1 in HME50 mock treated or exposed to LPA. *LPAR3 p=0.0302, *CTNNB1 p=0.0170, **LEF1 p=0.0052. Error bars represent SD over three biological replicates. **(e)** Expression trajectories of Ctnnb1 and Lpar3 genes in the luminal (green) and basal (red) populations. Normalized counts per million are plotted at each developmental time point. Error bars represent standard errors. **(f)**Expression trajectories of *Lef1* and *Tcf7* genes in the luminal (green) and basal (red) populations. Normalized counts per million are plotted at each developmental time point. Error bars represent standard errors.

Antibodies and dilutions used for fluorescence activated cell sorting (FACS).

Population Selection	Antigen	Isotype	Fluorophore/ Conjugate	Dilution	Company
Positive Selection	mouse Cd24	rat IgG2b, κ	Alexa Fluor® 488	1:500	BioLegend (San Diego, CA)
	mouse/rat Cd29	armenian hamster IgG	Alexa Fluor® 647	1:200	BioLegend (San Diego, CA)
Negative Selection (Lineage negative markers)	mouse Cd11b	rat IgG2b, κ	PE	1:500	BioLegend (San Diego, CA)
	mouse Cd45	rat IgG2b, κ	PE	1:500	BioLegend (San Diego, CA)
	mouse Ly-6G/Ly-6C	rat IgG2b, κ	PE	1:500	BioLegend (San Diego, CA)
	mouse Ter-119	rat IgG2b, κ	PE	1:500	BioLegend (San Diego, CA)
	mouse Cd31	rat IgG2a	PE	1:500	BioLegend (San Diego, CA)
	human Cd140a	mouse IgG2a, κ	PE	1:500	BD Biosciences (San Jose, CA)
Isotype Controls	rat IgG2b, κ	rat IgG2b, κ	Alexa Fluor® 488	1:500	BioLegend (San Diego, CA)
	armenian hamster IgG	armenian hamster IgG	Alexa Fluor® 647	1:200	BioLegend (San Diego, CA)
	rat IgG2b, κ	rat IgG2b, κ	PE	1:500	BD Biosciences (San Jose, CA)
	rat IgG2a, κ	rat IgG2a, κ	PE	1:500	BD Biosciences (San Jose, CA)

Supplementary Methods

Initial Validation of Sorted Populations

Because we were specifically interested in comparing the luminal and basal subtypes across the developmental time points, we attempted to maintain the same gates for each time point, omitting the third unidentified population during lactation and involution. However, due to differences in the intensity of the fluorescence labeled antibodies over time, we had to adjust the gates slightly for each sort **(Supplementary Figure S1)**. The enriched luminal and basal cell populations were collected in wash media at 4°C. Finally, to validate the sorted populations, we re-sorted 1,000 cells from each of the samples and verified that they were enriched in their respective $Lin^{-}Cd24^{+}CD29^{lo}$ and $Lin^{-}Cd24^{+}CD29^{hi}$ populations, and there was less than 2% cell cross contamination **(Supplementary Figure S1)**.

DNA, RNA and Protein Isolation and Quantitation

DNA, RNA, and protein were isolated from the same population of sorted cells using the AllPrep® DNA/RNA Micro Kit from QIAGEN (Germantown, MD). The protocol for animal and human cells provided with the kit was followed with a three minor additions: first, sorted cells were immediately pelleted and homogenized in the lysis buffer (Buffer RTL Plus with 1% β-mercaptoethanol (Bio-Rad, Hercules, CA)) after sorting to reduce RNA degradation, second, elution of DNA and RNA was done twice to increase the yield of nucleic acids, finally, the protein pellet was stored at -80°C in 80% acetone for downstream applications. For long-term storage, DNA was stored at -20°C and RNA was stored at -80°C. RNA was first treated with RNase-free recombinant DNaseI (Roche, Indianapolis, IN) to remove any contaminating DNA as recommended by Roche. Briefly, 1µl DNaseI, 1µl RNase OUT (Invitrogen, Carlsbad, CA) and the 10X incubation buffer were added to the RNA. The RNA was incubated at room temperature for 15 minutes, then at 37°C for 15 minutes. The reaction was stopped and the RNA was purified with the RNeasy MinElute Cleanup Kit (QIAGEN, Germantown, MD) as

described in the kit protocol. The final concentration of the RNA was determined by first staining diluted RNA with the Quant-iT^{TM} Ribogreen® RNA Assay Kit (Invitrogen, Carlsbad, CA) following the kit protocol and measuring the quantity with a NanoDrop 3300 Fluorospectrometer (Thermo Fisher Scientific, Waltham, MA). Because of the low RNA yield, the quality of RNA was analyzed with the Agilent RNA 6000 Pico Assay on the Agilent 2100 Bioanalyzer (Agilent Technologies, Santa Clara, CA). A total of 3ng of RNA was used to assess the RNA quality.

RNA-seq Data Quality Assessment and Normalization

Post-sequencing, the data were automatically processed through the web-based system termed WASP (Wiki-based Automated Sequence Processor) that was developed, and is maintained, at Einstein [1]. Aside from its use as a sample submission and laboratory information management system, WASP conducts automated primary data analysis of massively-parallel sequencing-based assays. The WASP pipeline (version 3.0.60, rev 6425) was specifically designed to process and align sequencing reads. The raw RNA-seq files (FASTQ files) automatically uploaded onto the system and the sequencing quality metrics were assessed using FastQC [2]. The WASP pipeline then trimmed the adapter sequences and aligned the reads against the reference mouse genome using the Genomic Short-read Nucleotide Alignment Program (GSNAP; version 2012-07-20) [3]. For the present study, the reference genome used was the National Center for Biotechnology Information (NCBI) Build 37.1 mouse genome obtained from the Reference Sequence (RefSeq) database [4,5]. The assignment of reads to genes was performed by htseq-count (v0.5.3p3), a component of the HTSeq package for analyzing high-throughput sequencing data [6]. The output of this pipeline was a samples (columns) by genes (rows) table with the total number of counts aligned to each gene and resulted in a sum of 37,991 mapped transcripts. Normalization was conducted in R v2.15.1 statistical software [7] using the edgeR and package available through Bioconductor [8]. The output data from the WASP pipeline were normalized following the documentation provided with the edgeR package.

Briefly, the total number of counts per million (CPM) were calculated for each sample by dividing the total number of reads by $1X10^6$. A scaling factor was then computed for each sample based on the total CPM, and these factors were used to calculate the library sizes. To avoid skewing of the data we removed genes with an extremely high number of reads (> $2X10^6$ reads) and low variability across the samples. Low expressed genes across the samples (genes that have less than 1CPM across 3 samples) were also removed. The normalization and filtering methods used left a total of 17,606 unique genes for subsequent analyses.

Principal Component Analysis

The prcomp function that is part of the *stats* package in R [9] was used to calculate the amount of variability present between the samples. We then used a linear analysis of variance (ANOVA) modeling approach similar to that previously described [10] to calculate the p-values for the association between each of the different technical and biological covariates in our analysis and the principal components detected. The biological covariates include the stage, time point, cell type, and condition (combined time point and cell type) (see **Supplementary Methods Table 1 below**). The technical covariates are the RNA was isolation batch (Batch), the lane on which the samples were run (Lane), the RNA amplification batch was conducted (RNAamp) and the FACS sorting batch (Sorting time).

Supplementary Methods Table 1: Profile of Samples Collected for Sorting and Biological Validation				
**Cell Population**	**Stage**	**Time Point**	**Condition**	**Replicates**
**Luminal**	Non-pregnant	Week 10	W10.L	6
	Pregnancy	Pregnancy day 3.5	P3.L	6
		Pregnancy day 14.5	P14.L	6
	Lactation	Lactation day 3	L3.L	6
		Lactation day 10	L10.L	6
	Involution	Involution day 2	Id2.L	6
	Non-pregnant	Involution week 4	Iw4.L	6
	Non-pregnant	Month 4	M4.L	6
**Basal**	Non-pregnant	Week 10	W10.B	6
	Pregnancy	Pregnancy day 3.5	P3.B	6
		Pregnancy day 14.5	P14.B	6
	Lactation	Lactation day 3	L3.B	6
		Lactation day 10	L10.B	5
	Involution	Involution day 2	Id2.B	6
	Non-pregnant	Involution week 4	Iw4.B	5
	Non-pregnant	Month 4	M4.B	6

Identification of Sample Outliers

We used two approaches to detect outlier samples based on their normalized transcription profiles. First, we calculated the correlation matrix of the pairwise comparisons using the Pearson correlations **(Supplementary Figure S2a)**. Comparison across biological replicates resulted in an $R^2 > 0.85$ across all conditions. Our clustering analysis revealed prominent clustering based on the cell subtypes. Of the 47 samples analyzed we detected one outlier from this analysis, one of the basal pregnancy day 14.5 samples clustered with the luminal population samples **(Supplementary Figure S2a, black arrows)**. Next, we performed a

principal component analysis on the dataset using the prcomp function that is part of the *stats* package in R [9] As with the correlation analysis, the samples clustered primarily based on the cell population. The same sample emerged as an outlier and was removed from the analysis **(Supplementary Figure S2b, black arrow).** The normalization was performed again and subsequent analyses were conducted using the remaining 46 samples. Hierarchical clustering and plotting of the correlation matrix using the heatmap.3 function in the GMD package [11].

Real-time PCR

RNAseq technical validation was performed on cDNA from the same three biological replicates from P3.5 and L3 basal populations used for RNAseq. A custom TaqMan® Array (Thermo Fisher Scientific, Waltham, MA) was designed with probes for 32 genes (including *18sRNA* and *Gapdh* as internal controls) in triplicate and qPCR was run on an ABI StepOnePlus instrument. Genes were selected based on RNAseq expression differences in the two populations and probe availability. Relative expression was calculated after normalization to 18sRNA and the fold change expression in L3 relative to P3.5. Two probes were eliminated from analyses because no data were acquired for any of the samples tested.

Primers for *LPAR1, LPAR2, LPAR3, LPAR4, LPAR5, LPAR6, CTNNB1, LEF1,* and *GAPDH* were designed using Primer 3 web tool, choosing amplicons producing 80–100 bp products and excluding amplification of non-specific products by analyzing their sequences against public databases (BLAST). Primers for *FOXA1, MUC1, ELF5, LFT* and *MGF-E8* were synthetized as previously reported [12]. For all conditions RNA was extracted using the RNeasy Mini Kit (Quiagen) and 5μg were reverse-transcribed with Superscript II and random hexamers (ThermoFisher, Fair Lawn, NJ) according to the manufacturer's instructions. For gene expression analysis we used SYBR green detection system (SYBR Green Master Mix, Life Technologies, Carlsbad, CA) on an ABI StepOnePlus instrument, according to manufacturer's cycling conditions. The relative abundances of the mRNAs for the genes of interest were

calculated after normalization against *GAPDH* (glyceraldehyde-3-phosphate dehydrogenase) mRNA levels as an internal control. The primers sequences used are listed in **Supplementary Methods Table 2**.

Supplementary Methods Table 2. Sequences of the human primers used for qRT-PCR.

**Gene**	**Fwd primer 5'-3'**	**Rev primer 5'-3'**
**LPAR1**	GACAAAGAAATGAGCGCCAC	GGTCATTGCTGTGAACTCCA
**LPAR2**	CTGCTCCTGGATGGTTTAGG	AGTACACAGCAGCATTGACC
**LPAR3**	GCCTTCCTCATCATGGTTGT	GTATGCGGAGACAAGACGTT
**LPAR4**	TGCAAGATCTCTGGAACTGC	TAGACAATGGCCAGGAAACG
**LPAR5**	GTTAGCCAACAGCTCCTCAA	AAGCTGTAGACCACCAAGTG
**LPAR6**	CTTCACAACACGGAATTGGC	ATGCTTCCGTACATGTTGGT
**CTNNB1**	AAGCCGGCTATTGTAGAAGC	GACCTTCCATCCCTTCCTGTTT
**Lef1**	ACCCATCCCGAGAACATCAA	TCTGATGGGATGTGTGACGG
**GAPDH**	TCAAGAAGGTGGTGAAGCAG	CGCTGTTGAAGTCAGAGGAG

Biological Validation of Cell Subtypes with Publically Available Datasets

The luminal and basal populations were biologically validated using a gene set derived from enriched mouse and human mammary epithelial populations [13,14]. The *Biobase* [15], *GEOquery* [16] and *limma* packages were used in R to identify DE genes in this dataset. The normalized mouse and human datasets (GEO accession GSE19446 and GSE16997, respectively) were downloaded from the NCBI Gene Expression Omnibus (GEO) database [17,18]. The data were log 2 transformed and DE genes were identified by making pairwise comparisons between the luminal and basal cell populations. Mature luminal genes were identified by comparing the mature luminal samples versus the basal samples. Luminal progenitor samples were identified by comparing the luminal progenitors to the basal samples. Basal specific genes were identified by comparing the basal samples to both the mature luminal and luminal progenitor samples. The same threshold used for our dataset was applied to this dataset to generate a statistically significant DE geneset (fold change > 2 and FDR < 1%). Since the luminal epithelial samples in our dataset contain a mixture of luminal progenitors and mature luminal cells, we combined the

mature luminal and luminal progenitor gene data of the published sets for our enrichment analysis. The above analyses were done for both the human and mouse datasets. The human genes were then converted to their mouse orthologs by using the annotations available through Ensembl [19]. Of the 1,816 luminal-specific genes in the human, 1,332 (73.3%) had annotated moues orthologs. Of the 1,544 basal-specific genes, 947 (61.3%) had annotated mouse orthologs. We used a hypergeometric distribution test to test for the enrichment of our geneset in the Lim et al mouse and human genesets [13]. This same method was used to calculate enrichment in the β-catenin mammary tumor models from GSE43825 [20].

Statistical Methods for the Identification of Spatially and Temporally Expressed Genes

ANOVA was used to identify genes that were significantly differentially expressed in at least one condition (**see Supplementary Methods Table 1**), defined as "expressed" genes, as previously described [21]. Based on our PCA analysis we were confident that the technical covariates did not significantly contribute to the overall variability between the samples, thus we did not include them in the linear model for ANOVA. After correcting for multiple testing using the Benjamini & Hochberg false discovery rate (FDR) method [22], we set a statistical threshold of less than 0.01 (1%) to define the spatially and temporally "expressed" genes. This set of "expressed" genes was then used for subsequent analyses using edgeR package, available through Bioconductor in R, to determine cell type and time point specific genes [8]. Correction for multiple testing was performed using the Benjamini & Hochberg false discovery rate (FDR) method [22] and set a threshold of an absolute fold change > 2 and an FDR < 1%.

Reactome, Gene Ontology and KEGG Enrichment Analysis

The GOseq package [23] was used to test for enrichment of our gene sets in functional groups based on their annotation defined by the Reactome database [24], Gene Ontology (GO) Consortium [25], or KEGG database [26]. We tested specifically for enrichment in the Biological Processes category and set a p-value cutoff of 0.05. Functional interaction network analysis

was conducted using the Reactome FI pug-in in cytoscape [27].

Gene Set Enrichment Analysis

Using transcriptional profiling of basal and luminal cells during postnatal mammary gland development, we generated stage specific gene lists. Individual gene lists were converted for use as gene sets for Gene Set Enrichment Analysis [28]. Using GSEA and a published database of mouse mammary tumor models [29], we queried individual mouse mammary tumor models for enrichment of basal or luminal stage specific gene sets.

Immunostaining of Tissue Sections

The mouse mammary inguinal gland was fixed in 10% formalin and paraffin embedded, 5 micrometer slices were sectioned and added onto slides. Slides with tissue sections were baked at 60°C for 1 hour, deparaffinized in xylene (twice for 10 minutes each time) and rehydrated in a series of 2 minute washes in ethanol (100%, 95%, 80%, 70%) followed by two washes in distilled water. Antigen retrieval was performed using the Vector® Antigen Unmasking Solution (H-3300, Vector Laboratories, Burlingame, CA) for 20 minutes and then cooled on the bench for 30 minutes in the buffer solution. The tissue was blocked in 8% goat serum/ in TBS for 60 minutes at room temperature. Sections were then incubated in the anti-CK14 (Covance, Dedham, MA), anti-LPAR3 (LifeSpan BioSciences, Inc., Seattle, WA), anti-LPAR1 (LifeSpan BioSciences, Inc., Seattle, WA) and anti-beta-catenin (abcam, Cambridge, MA) antibodies overnight at 4°C. These antibodies were used at a dilution of 1:1000, 1:200, 1:400 and 1:400 respectively. Secondary detection was performed using anti-mouse Alexa 488 dye (Invitrogen), Alexa 594 dye (Invitrogen) and Alexa 680 dye (Invitrogen) at a dilution of 1:200, 1:400 and 1:400 respectively. Primary and secondary antibodies were diluted in 5% goat serum/TBS. The tissues were counterstained with DAPI (4',6-diamidino-2-phenylindole) (Chroma Technologies, Bellows Falls, VT). All images were acquired using a Leica SP2-AOBS confocal microscope (Leica Microsystems, Buffalo Grove, IL) and quantitation done using Volocity 3D Image Analysis

Software (PerkinElmer, Waltham, MA).

For immunofluorescence, both control and LPA treated HME50 cells were grown in tissue culture petri dish containing glass cover slips. After exposure to LPA for 72h, the cover slips were removed and fixed using 4% Paraformaldehyde (PFA) for 15min at room temperature (RT). This was then replaced by 0.1% Triton (dissolved in PBS) for 10min, RT. After washing 3x with PBS the coverslips were blocked with 3% goat serum for 30 min, RT. For simultaneous identification of basal and luminal cells, the cells were incubated for 1h with primary antibody mixture containing mouse monoclonal to Cytokeratin18 (luminal specific) and Keratin14 Polyclonal chicken antibody (basal specific). This was followed by 3 washes with PBS and exposure of the slides to secondary antibodies for 1hr containing goat anti chicken, Alexa Fluor 488 (green) and goat anti mouse, Alexa Fluor 555 (red). The coverslips were washed and dehydrated with ethanol at room temperature – 70% for 3min; 90% for 3min; 100% for 3min. The coverslips were air dried for 1h. Mounting medium (Life Technologies, Carlsbad, CA) (10µl) with DAPI (blue) was placed on a slide and the coverslips were inverted on to it. The slides were visualized under Axiovert 200 inverted fluorescent microscope and the image captured using Case Data Manager 6.0 software (Applied Spectral Imaging, ASI Carlsbad, CA). The images were then analyzed using Image J software.

Cell fractionation and western blotting

Cell fractionation was performed as per the manufacturer's instructions using the Qiagen, CA, USA kit. Protein estimation for each of the cell fractions were carried out using BCA kit as per the manufacturer's protocol (Pierce Thermo Scientific, IL, USA). Mouse monoclonal to Cytokeratin 18 (K18) (primary antibody) was purchased from Abcam, MA, USA. Keratin 14 (K14) (primary antibody) Polyclonal Chicken Antibody was purchased from Covance, NJ, USA. Purified Mouse Anti- β – catenin (primary antibody) was purchased from BD Biosciences, USA.

Sodium dodecyl sulphate - polyacrylamide gel electrophoresis (SDS-PAGE) was conducted using 10% (w/v) separating gel. 50µg/ml proteins samples of membrane and nuclear fraction of LPA treated and control cells were separated by denaturing SDS-PAGE and then transferred to nitrocellulose membranes. The membranes were blocked with 5% (w/v) skimmed milk. The membrane fractions were incubated with Mouse Anti-β-catenin (1/2000) and Mouse monoclonal Anti –α–tubulin (1/1000) and the nuclear fractions were incubated with Mouse Anti-β-catenin (1/2000) and Rabbit monoclonal Histone H3 (1/2000) for 1h. After being washed, the membranes were incubated with goat anti-mouse HRP conjugate and goat anti-rabbit HRP conjugate (1/2000) for 1h. Proteins were visualized by addition of substrate (luminol).

Quantification of bands intensities was carries out using ImageJ [30]. After subtraction of background intensities the β-catenin specific expression was normalized against its respective control (Histone H3 for the nuclear fraction andα–tubulin for the membrane fraction); plots and statistical analysis were carried out using GraphPad Prism version 6 for Windows (GraphPad Software, La Jolla California USA, www.graphpad.com).

References

1 Core, C. E. T. a. C. G. *Wiki-based Automated Sequence Processor (WASP)*, <http://wasp.einstein.yu.edu/> (

2 Andrews, S. *FastQC*, <http://www.bioinformatics.bbsrc.ac.uk/projects/fastqc> (

3 Wu, T. D. & Nacu, S. Fast and SNP-tolerant detection of complex variants and splicing in short reads. *Bioinformatics* **26**, 873-881, doi:10.1093/bioinformatics/btq057 (2010).

4 Mouse Genome Sequencing, C. *et al.* Initial sequencing and comparative analysis of the mouse genome. *Nature* **420**, 520-562, doi:10.1038/nature01262 (2002).

5 Pruitt, K. D., Tatusova, T., Brown, G. R. & Maglott, D. R. NCBI Reference Sequences (RefSeq): current status, new features and genome annotation policy. *Nucleic Acids Res* **40**, D130-135, doi:10.1093/nar/gkr1079 (2012).

6 Anders, S. *HTSeq: Analysing high-throughput sequencing data with Python*, <http://www-huber.embl.de/users/anders/HTSeq/> (

7 R Core Team, R: A language and environment for statistical computing. R Foundation for Statistical Computing. (Vienna, Austria, 2016).

8 Robinson, M. D., McCarthy, D. J. & Smyth, G. K. edgeR: a Bioconductor package for differential expression analysis of digital gene expression data. *Bioinformatics* **26**, 139-140, doi:10.1093/bioinformatics/btp616 (2010).

9 Walter, W., Sanchez-Cabo, F. & Ricote, M. GOplot: an R package for visually combining expression data with functional analysis. *Bioinformatics* **31**, 2912-2914, doi:10.1093/bioinformatics/btv300 (2015).

10 Teschendorff, A. E., Zhuang, J. & Widschwendter, M. Independent surrogate variable analysis to deconvolve confounding factors in large-scale microarray profiling studies. *Bioinformatics* **27**, 1496-1505, doi:10.1093/bioinformatics/btr171 (2011).

11 Zhao, X., Valen, E., Parker, B. J. & Sandelin, A. Systematic clustering of transcription start site landscapes. *PLoS One* **6**, e23409, doi:10.1371/journal.pone.0023409 (2011).

12 Shehata, M. *et al.* Phenotypic and functional characterisation of the luminal cell hierarchy of the mammary gland. *Breast Cancer Res* **14**, R134, doi:10.1186/bcr3334 (2012).

13 Lim, E. *et al.* Transcriptome analyses of mouse and human mammary cell subpopulations reveal multiple conserved genes and pathways. *Breast Cancer Res* **12**, R21, doi:10.1186/bcr2560 (2010).

14 Lim, E. *et al.* Aberrant luminal progenitors as the candidate target population for basal tumor development in BRCA1 mutation carriers. *Nature medicine* **15**, 907-913, doi:10.1038/nm.2000 (2009).

15 Gentleman, R. C. *et al.* Bioconductor: open software development for computational biology and bioinformatics. *Genome Biol* **5**, R80, doi:10.1186/gb-2004-5-10-r80 (2004).

16 Davis, S. & Meltzer, P. S. GEOquery: a bridge between the Gene Expression Omnibus (GEO) and BioConductor. *Bioinformatics* **23**, 1846-1847, doi:10.1093/bioinformatics/btm254 (2007).

17 Barrett, T. *et al.* NCBI GEO: archive for functional genomics data sets--update. *Nucleic Acids Res* **41**, D991-995, doi:10.1093/nar/gks1193 (2013).

18 Edgar, R., Domrachev, M. & Lash, A. E. Gene Expression Omnibus: NCBI gene expression and hybridization array data repository. *Nucleic Acids Res* **30**, 207-210 (2002).

19 Flicek, P. *et al.* Ensembl 2012. *Nucleic Acids Res* **40**, D84-90, doi:10.1093/nar/gkr991 (2012).

20 Moumen, M. *et al.* Myc is required for beta-catenin-mediated mammary stem cell amplification and tumorigenesis. *Molecular cancer* **12**, 132, doi:10.1186/1476-4598-12-132 (2013).

21 Pavlidis, P. Using ANOVA for gene selection from microarray studies of the nervous system. *Methods* **31**, 282-289, doi:Doi 10.1016/S1046-2023(03)00157-9 (2003).

22 Benjamini, Y. & Hochberg, Y. Controlling the False Discovery Rate - a Practical and Powerful Approach to Multiple Testing. *J Roy Stat Soc B Met* **57**, 289-300 (1995).

23 Young, M. D., Wakefield, M. J., Smyth, G. K. & Oshlack, A. Gene ontology analysis for RNA-seq: accounting for selection bias. *Genome Biol* **11**, R14, doi:10.1186/gb-2010-11-2-r14 (2010).

24 Ligtenberg, W *reactome.db: A set of annotation maps for reactome*. R package version 1.54.1.

25 Ashburner, M. *et al.* Gene ontology: tool for the unification of biology. The Gene Ontology Consortium. *Nat Genet* **25**, 25-29, doi:10.1038/75556 (2000).

26 Carlson, M *KEGG.db: A set of annotation maps for KEGG*. R package version 3.2.2.

27 Wu, G., Feng, X. & Stein, L. A human functional protein interaction network and its application to cancer data analysis. *Genome Biol* **11**, R53, doi:10.1186/gb-2010-11-5-r53 (2010).

28 Subramanian, A. *et al.* Gene set enrichment analysis: a knowledge-based approach for interpreting genome-wide expression profiles. *Proc Natl Acad Sci U S A* **102**, 15545-15550, doi:10.1073/pnas.0506580102 (2005).

29 Hollern, D. P. & Andrechek, E. R. A genomic analysis of mouse models of breast cancer reveals molecular features of mouse models and relationships to human breast cancer. *Breast Cancer Res* **16**, doi:10.1186/bcr3672 (2014).

30 Schneider CA, Rasband WS, Eliceiri KW. NIH Image to ImageJ: 25 years of image analysis. *Nat Methods. Jul;9(7):671-5* (2012).
